# The myocardial regenerative potential of three-dimensional engineered cardiac tissues composed of multiple human iPS cell-derived cardiovascular cell lineages

**DOI:** 10.1038/srep29933

**Published:** 2016-07-20

**Authors:** Hidetoshi Masumoto, Takeichiro Nakane, Joseph P. Tinney, Fangping Yuan, Fei Ye, William J. Kowalski, Kenji Minakata, Ryuzo Sakata, Jun K. Yamashita, Bradley B. Keller

**Affiliations:** 1 Kosair Charities Pediatric Heart Research Program, Cardiovascular Innovation Institute, University of Louisville, Louisville, Kentucky, The United States of America; 2 Department of Cell Growth and Differentiation, Center for iPS Cell Research and Application (CiRA), Kyoto University, Kyoto, Japan; 3 Department of Cardiovascular Surgery, Kyoto University Graduate School of Medicine, Kyoto, Japan; 4 Department of Pediatrics, University of Louisville School of Medicine, Louisville, Kentucky, The United States of America

## Abstract

Human induced pluripotent stem cells (hiPSCs) are a robust source for cardiac regenerative therapy due to their potential to support autologous and allogeneic transplant paradigms. The *in vitro* generation of three-dimensional myocardial tissue constructs using biomaterials as an implantable hiPSC-derived myocardium provides a path to realize sustainable myocardial regeneration. We generated engineered cardiac tissues (ECTs) from three cellular compositions of cardiomyocytes (CMs), endothelial cells (ECs), and vascular mural cells (MCs) differentiated from hiPSCs. We then determined the impact of cell composition on ECT structural and functional properties. *In vitro* force measurement showed that CM+EC+MC ECTs possessed preferential electromechanical properties versus ECTs without vascular cells indicating that incorporation of vascular cells augmented tissue maturation and function. The inclusion of MCs facilitated more mature CM sarcomeric structure, preferential alignment, and activated multiple tissue maturation pathways. The CM+EC+MC ECTs implanted onto infarcted, immune tolerant rat hearts engrafted, displayed both host and graft-derived vasculature, and ameliorated myocardial dysfunction. Thus, a composition of CMs and multiple vascular lineages derived from hiPSCs and incorporated into ECTs promotes functional maturation and demonstrates myocardial replacement and perfusion relevant for clinical translation.

Stem cell-based cardiac regeneration is a rapidly expanding paradigm with the expectation of delivering novel therapeutic approaches for severe cardiac disorders resistant to current therapies^1^,^2^,^3^,^4^,^5^. The *in vitro* formulation of three-dimensional (3D) organized and implantable myocardial tissue constructs has emerged as a robust method to realize myocardial replacement in preclinical models^6^,^7^,^8^,^9^. The acceleration of structural and functional *in vitro* maturation, optimized *in vivo* survival, and functional integration of human cell-derived engineered cardiac tissues (ECTs) to failing recipient myocardium remain major requirements for clinical translation. While much of the focus has been on the incorporation of cardiomyocyte (CM) lineages into ECTs, non-myocytes including vascular cell lineages serve pivotal roles during myocardial development and in the generation and remodeling of bioengineered tissue architecture^7^,^8^,^10^. Engineered constructs that include these multiple lineage constructs are more likely to replicate the dynamic organization and function of native myocardium.

Among the stem cell types, pluripotent stem cells [Embryonic stem cells (ESCs)/induced pluripotent stem cells (iPSCs)] possess great potential for cardiac regeneration due to their capacity for robust *in vitro* expansion followed by directed differentiation into most somatic cell lineages including CMs or other vascular cell types^11^,^12^. The generation of iPSCs by reprogramming somatic cells with genes regulating pluripotency may resolve the ethical and immunogenic issues associated with the clinical application of ESCs.

We have investigated multiple compositions of ECTs generated from immature chick or rat fetal/neonatal heart cells^13^,^14^,^15^ to validate preclinical cardiac repair paradigms. Furthermore, we also have reported that co-existence of CMs and vascular cell lineages within mouse ESC-engineered cell sheet structure augmented cell sheet function^10^, and the transplantation of cell sheets engineered from human iPSC (hiPSC)-derived multiple cardiac lineages (cardiac tissue sheets) brought functional recovery and myocardial regeneration on a rat myocardial infarction (MI) model^8^. We hypothesized that ECTs composed by human iPSC (hiPSC)-derived cardiovascular cells hold promise to realize an ideal 3D structure for clinical application. In the present study, we utilize a strategy that identified an optimal composition of multiple cardiovascular cell populations derived from hiPSCs to generate ECT grafts with improved tissue maturation and with excellent post-implantation survival, perfusion, and contribution to functional recovery relevant to clinical application.

## Results

**Figure 1:**
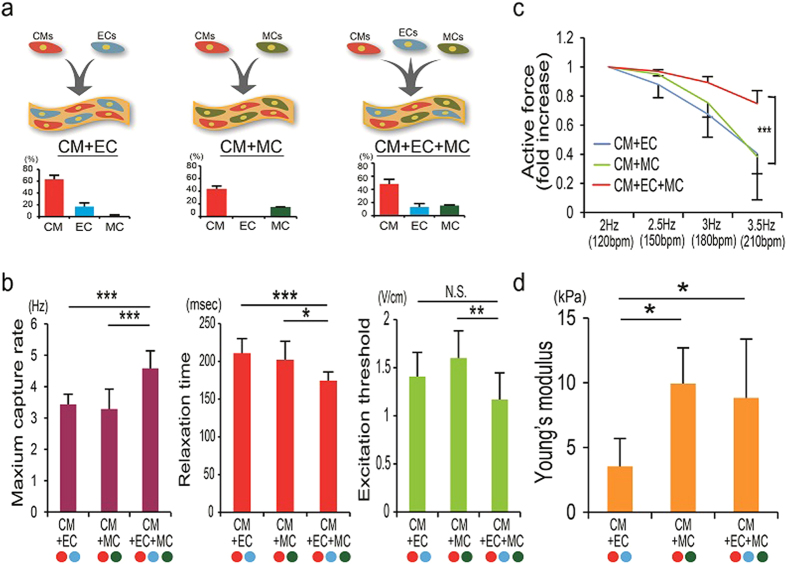
Electromechanical properties of hiPSC-ECTs. (**a**) Schematic diagrams for composition of 3 types of ECTs (upper) containing cardiomyocytes (CM), endothelial cells (EC), and/or mural cells (MC) and the proportions of each cell type used to generate ECTs (lower). Groups sizes were n = 8 (CM+EC), n = 7 (CM+MC), and n = 12 (CM+EC+MC). (**b**–**d**) Results of contractile force measurements [n = 8 (CM+EC), n = 7 (CM+MC) and n = 12 (CM+EC+MC)]. (**b**) Maximum capture rate (left), relaxation time (center) and excitation threshold (right) (**c**) Transition of active force according to the increase of pacing frequency (from 2Hz to 3.5Hz). (**d**) Young’s modulus. NS, not significant; *P < 0.05, **P < 0.01, ***P < 0.001.

We optimized our previously reported hiPSC cardiovascular cell induction protocol to induce CMs along with endothelial cells (ECs)^8^ which resulted in the efficient induction of cardiac troponin-T (cTnT)^+^ CMs (61.8 ± 8.0% of total cells) and vascular endothelial (VE)-cadherin (CD144)^+^ ECs (19.4 ± 9.1%) with little co-induction of platelet-derived growth factor receptor beta (PDGFRβ; CD140b)^+^ vascular mural cells (MCs) (1.7 ± 2.0%) by day 15 (d15; n = 26) (Supplementary Fig. 1; CM+EC protocol). Wnt3a treatment significantly increased CM and EC induction and decreased MC induction (Supplementary Fig. 2). We generated ECTs composed of cells harvested on d15, collagen I and Matrigel. ECTs displayed spontaneous beating after 3 to 4 days in culture (hiPSC-ECT with CMs and ECs; CM+EC) (Fig. 1a, Supplementary Fig. 3a and Supplementary Video 1). Human iPSC-derived ECTs underwent rapid gel compaction which facilitated continued nutrient delivery during construct maturation^16^ (Supplementary Fig. 4).

To explore the potential for vascular mural cells (MCs) to further accelerate ECT formation and maturation, we expanded the vascular lineage composition and revised our ECT composition to include MCs which are abundant in maturing and adult muscles, including the heart, and facilitate tissue growth, physiology^17^,^18^,^19^ and structural integrity^7^,^8^,^10^. We employed 2 additional hiPSC differentiation protocols. The primary MC protocol is a modification of a previously reported method^20^,^21^ that preferentially induces MCs without CMs or ECs (Supplementary Fig. 1) and resulted in 74.4 ± 8.4% PDGFRβ^+^ MCs (n = 42). A secondary CM+MC protocol is modified from our previous report^22^ to preferentially induce CMs and MCs without ECs (Supplementary Fig. 1). We combined cells from these 3 induction protocols to adjust final MC ratios as 5 to 25% of total cells, and generated 2 additional ECT classes: ECTs with CMs, ECs and MCs (CM+EC+MC), and ECTs with CMs and MCs but without ECs (CM+MC) (Fig. 1a and Supplementary Fig. 3a). The cellular components of TRA-1-60-positive undifferentiated hiPSCs within cell mixtures used for each ECT class were 4.1 ± 3.7% in CM+EC (n = 17), 5.9 ± 0.9% in CM+MC (n = 7) and 5.9 ± 2.8% in CM+EC+MC (n = 33), respectively.

We assessed ECT electromechanical properties and functional maturation using a custom intact muscle test system (Supplementary Fig. 3b,c). Active force generation trended higher in the CM+EC ECT group at 2Hz/5V pacing, consistent with a higher CM ratio in CM+EC (63.1 ± 7.0%) compared to other 2 types (CM+EC+MC: 48.3 ± 6.8%, P < 0.001; CM+MC: 43.6 ± 4.7%, P < 0.0001) (Supplementary Fig. 5a). Interestingly, there was no direct correlation between CM ratio and active force (P = 0.248), highlighting the important contributions of non-CM subpopulations (Supplementary Fig. 5b). The highest active stress generated by hiPSC-ECTs was 1.49 mN/mm^2^ (average of 0.62 mN/mm^2^), comparable to human pediatric myocardium (1–1.5 mN/mm^2^), but less than adult myocardium (15–30 mN/mm^2^)^23^. Next, we found that the paced maximum capture rate (MCR) was significantly higher in CM+EC+MC (consistently greater than 240 bpm) compared to other ECT types (Fig. 1b). We also found that relaxation time (RT)^24^ (Supplementary Fig. 6b) was shortest in CM+EC+MC, which would facilitate higher MCR (Fig. 1b). In CM+EC+MC, there was a positive correlation between MC ratio and MCR (R^2^ = 0.433, P < 0.05), and negative correlation between MC ratio and RT (R^2^ = 0.669, P < 0.01) (Supplementary Fig. 6) indicating ratio-dependent contribution of MCs to MCR. We also noted that the excitation threshold (ET) required to induce ECT contraction was significantly lower in CM+EC+MC compared to CM+MC, and lower but not significantly different than CM+EC (Fig. 1b). We next assessed active force production at frequencies higher than 2 Hz (120 bpm). Approximately 75% of active force was maintained at 3.5 Hz (210 bpm) in CM+EC+MC, whereas active force in CM+EC or CM+MC at 3.5 Hz decreased to approximately 40% of baseline 2 Hz-force (Fig. 1c). These results indicate that ECTs incorporating both MCs and ECs have the potential for coupling to higher *in vivo* recipient beat rates after implantation without decreasing the active force production. Thus, ECTs composed of CM+EC+MC demonstrated excellent electromechanical properties compared to other ECT classes.

We further examined whether tissue stiffness, an important feature of tissue maturation and function, could be altered by vascular cell incorporation. In the native heart, passive force is affected by tissue length according to Hooke’s law (law of elasticity)^25^ and tissue elongation increases active force according to Frank-Starling mechanisms^26^. We measured and plotted passive stresses at increasing lengths (up to 25%) from slack length and found that the passive stress-length relationship was consistent with Hooke’s law for all ECT compositions (Supplementary Fig. 7a) (average R^2^ value: 0.922 in CM+EC, 0.885 in CM+MC, 0.882 in CM+EC+MC)^25^. We found that Young’s modulus were higher in ECTs with MCs (CM+MC and CM+EC+MC) compared to those without MCs (CM+EC), indicating that MCs contribute to produce higher tissue stiffness (Fig. 1d). The highest Young’s modulus in CM+EC+MC reached over 17 kPa (average of 8.8kPa) which is comparable to reported normal cardiac muscle tissue stiffness (10–15 kPa)^27^. Active stress-length relationships for all ECT compositions were consistent with a positive Frank-Starling relationship similar to the native heart (average R^2^ value: 0.972 in CM+EC, 0.961 in CM+MC, 0.973 in CM+EC+MC) indicating that ECTs recapitulate a fundamental property of native cardiac muscle (Supplementary Fig. 7b)^25^. We examined the effect of MCs on ECT remodeling. Gel compaction rate was higher in ECTs with MCs compared to those without MCs (Supplementary Fig. 4).

To assess Ca^2+^ channel and sarcoplasmic reticulum (SR) maturation of hiPSC-derived CMs included in each class of ECTs, we performed force measurement analyses under various Ca^2+^ concentrations and administration of caffeine. There was a tendency (not significant) of immature SR pattern in ECTs without ECs (CM+MC) compared to ECTs with ECs (CM+EC and CM+EC+MC) in response to external Ca^2+^ concentration (Supplementary Fig. 8a)^28^ or caffeine (5 mM) to increase SR ryanodine channel open state (Supplementary Fig. 8b)^16^. However, the Ca^2+^ EC50 (half maximal effective concentration) values of our ECTs (Supplementary Fig. 8a) (around 1 mM) were much lower than those reported as those for adult human muscle strips (around 3.0 mM)^28^, consistent with a greater reliance of immature myocardium on sarcolemmal Ca^2+^ influx rather than Ca^2+^ release from SR for contraction, similar to reported human pluripotent stem cell-derived CMs^16^. Thus, although there are obvious advantages to the CM+EC+MC ECT composition relevant to electromechanical function, we noted no clear preferential effect on the maturation of individual CM Ca^2+^ channel and SR function indicating co-existence of vascular cells within 3D tissue structure compensated the immaturity of individual CMs.

**Figure 2:**
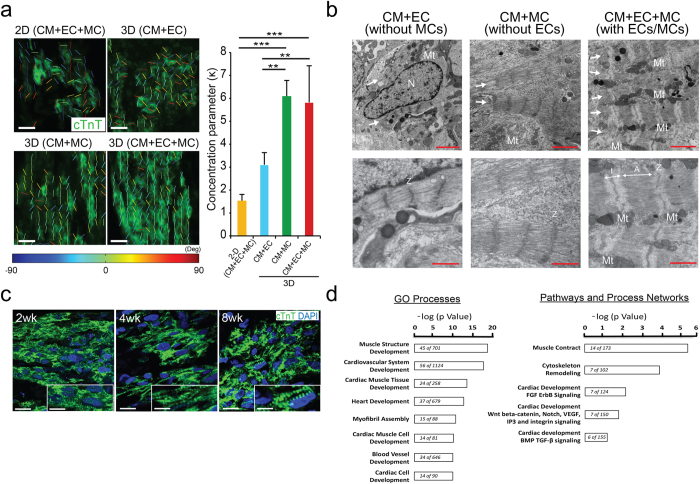
Structural and functional maturation of hiPSC-ECTs including vascular cells. (**a**) Representative alignment analysis using cTnT-stained images after 2-dimensional (2D) and 3-dimensional (3D) culture (left) (see Supplementary Fig. 8 for whole images of nuclei and myofiber) for 3 types of ECTs containing cardiomyocytes (CM), endothelial cells (EC), and/or mural cells (MC). Calculated concentration parameter (κ), an index of that is related to myofiber alignment, for each culture condition (right) [n = 3 (2D) and 5 (each 3D)]. cTnT, cardiac troponin T; Deg, degree. (**b**) Representative Transmission electron microscopic images for each type of ECT cultured 4 weeks. Lower panels indicate higher magnification images. Arrows indicate myofibers. N, nucleus; Mt, mitochondria; I, I-band; A, A-band; Z, Z-line. (**c**) Representative cTnT immunostaining of ECTs (CM+EC+MC) cultured *in vitro* for different periods. Inset shows single CM image showing sarcomeric structure. 2/4/8 wk, cultured *in vitro* for 2, 4, or 8 weeks, respectively. DAPI, 4, 6 diamidino-2-phenylindole. (**d**) Representative gene processes significantly regulated by the addition of mural cells (CM+EC+MC versus CM+EC) in hiPSC-ECTs. Gene ontology processes (left), and pathways and process networks (right). Scale bars represent significance expressed as the –log p Value and values within each bar represent the number of altered genes and total genes in each network. Scale bars: 20 μm in (**a**), (**c**) (main panels), 10 μm in (**c**) (inset), 2 μm in (**b**) (upper panels) and 1 μm in (**b**) (lower panels). **P < 0.01, ***P < 0.001.

Next, we investigated structural mechanisms that could account for the functional advantages of incorporating vascular cells into ECTs. We analyzed myofiber alignment and found that ECTs containing MCs had increased alignment as quantified by increased concentration parameter (κ) (Fig. 2a) and reduced circular standard deviation (Supplementary Fig. 9) consistent with increased CM alignment parallel to the ECT long axis. As expected, tissue maturation within 3D ECT constructs was associated with greater cellular alignment compared to 2D culture. We further noted that sarcomeric structural maturation in individual CM was more pronounced in CMs within ECTs containing both MCs and ECs (CM+EC+MC). ECTs with both ECs and MCs demonstrated parallel myofibers with clear Z-line, I-band and A-band along with mitochondria localized between myofibers, a representative feature of mature myocytes not consistently present in other ECT classes (Fig. 2b). Sarcomeric structural maturation was more prominent following longer *in vitro* culture up to 8 weeks in ECTs including ECs and MCs (Fig. 2c). Thus, we noted that vascular incorporation was associated with *in vitro* tissue remodeling that improves biophysical properties including better cellular alignment and increased structural maturation of individual CM. We further examined whether vascular cell incorporation altered gene expression profiling in ECTs. RNA sequencing analysis for ECTs after 14 days of *in vitro* culture revealed the inclusion of MCs resulted in the activation of multiple gene ontology pathways consistent with accelerated tissue maturation (Fig. 2d), which might have contributed to observed increased ECT structural and functional maturation.

**Figure 3:**
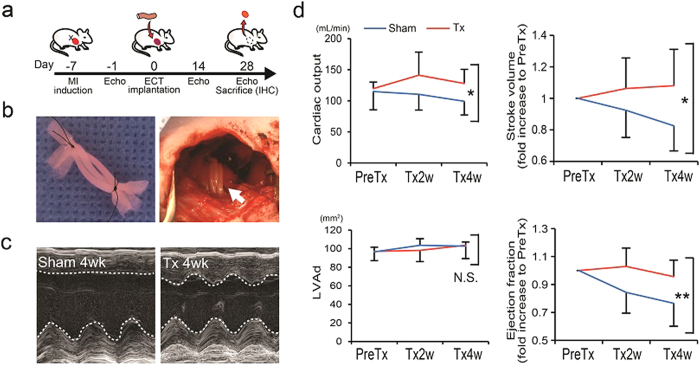
Cardiac implantation of hiPSC-ECTs on the infarcted rat heart and functional recovery. (**a**) Schematic timeline of rat surgery. MI, myocardial infarction; Echo, echocardiogram; IHC, immunohistochemistry. (**b**) Three hiPSC-ECTs bundled and prepared for implantation (left). ECTs implanted onto the heart surface at infarction site (right) (arrow). (**c,d**) Results of echocardiogram. (**c**) Representative M-mode images for sham-operated (left) and ECT-implanted (right) rats 4 weeks after surgery. Tx, ECT-implanted. (**d**) Results of B-mode echocardiogram [n = 6 (Tx) and 5 (sham)]. Cardiac output (mL/min) (left upper), stroke volume (fold increase to PreTx) (right upper), left ventricular end diastolic area (LVAd; mm^2^) (left lower) and ejection fraction (fold increase to PreTx) (right lower). PreTx, before treatment; Tx2w/4w, 2/4 weeks after treatment. *P < 0.05. **P < 0.01. N.S., Not significant.

**Figure 4:**
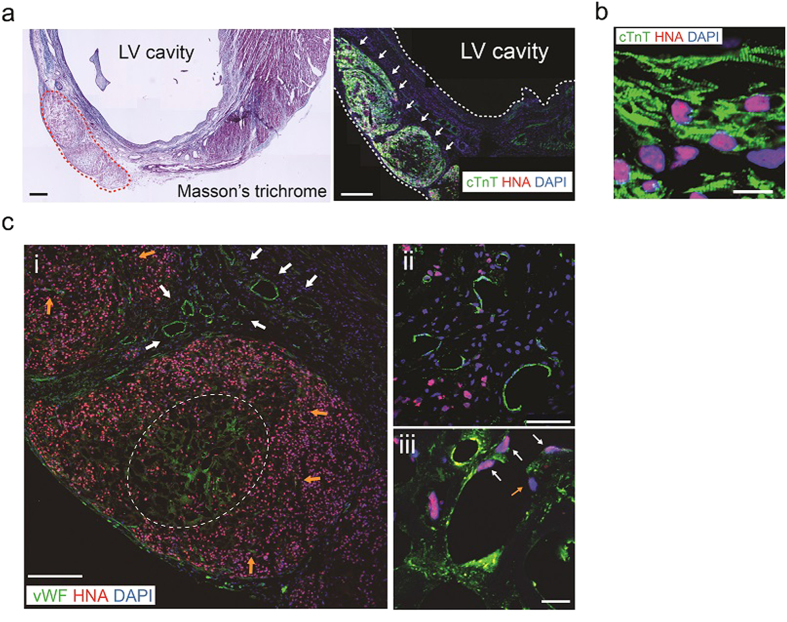
Myocardial regeneration and post-implantation *in vivo* reorganization of hiPSC-ECTs. (**a**–**c**) Left ventricular (LV) histology 4 weeks after implantation of hiPSC-ECTs (including 5% MCs). (**a**) Masson’s trichrome staining (left). Red dotted line indicates engrafted area. cTnT and HNA (human cell marker) double immunostaining (right). Arrows indicate engrafted myocardium. White dotted lines indicate LV border. LV, left ventricle; HNA, human nucleic antigen. (**b**) Higher magnification of cTnT and HNA double immunostaining showing sarcomeric structure. (**c**) vWF (endothelial cell marker) and HNA double immunostaining. (i) Lower magnification image. White arrows indicate promoted capillary formation around grafted tissue. Orange arrows indicate penetrating vasculature. White dotted line indicates vasculature in the center of grafted tissue. (ii) and (iii) Higher magnification images. (ii) Prominent host-derived (HNA^−^) vascular formation around regenerated myocardium. (iii) chimeric vasculature composed of both host (HNA^−^; orange arrow) and graft (HNA^+^; white arrows) vascular cells. vWF, von Willebrand factor. Scale bars: 500 μm in (**a**), 200 μm in (**c**) (i), 50 μm in (**c**) (ii), 20 μm in (**c**) (iii), and 10 μm in (**b**).

**Figure 5:**
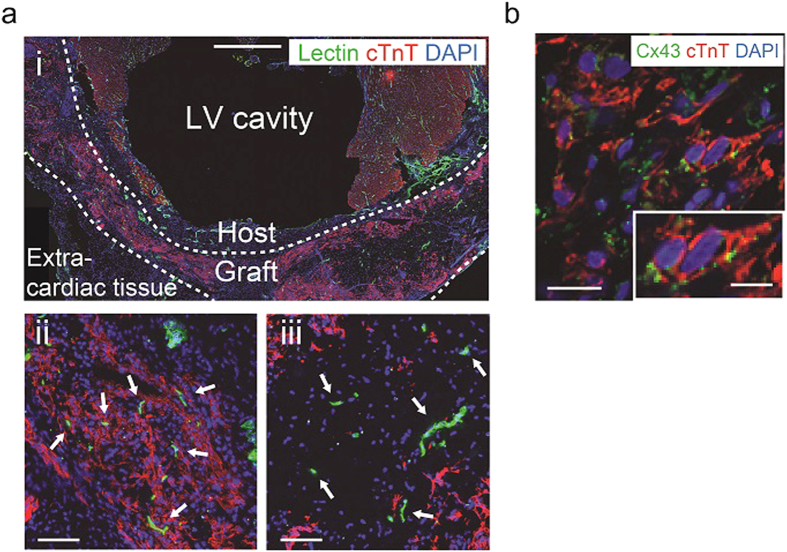
Functional coupling of implanted hiPSC-ECTs. (**a**) cTnT immunostaining for lectin (represents perfused vasculature)-perfused rat heart 4 weeks after implantation of hiPSC-ECTs (including 16% MCs). (i) Lower magnification image. (ii) and (iii) Higher magnification images. Perfused vasculature among (ii) and at central area (iii) of regenerated myocardium (arrows). (**b**) Cx43 (Connexin 43) and cTnT double immunostaining for regenerated myocardium. Scale bars: 1mm in (**a**) (i), 100 μm in (**a**) (ii, iii), 20 μm in (**b**) (main panel), and 10 μm in (**b**) (inset).

Finally, considering the results that ECTs including all 3 cardiovascular lineages (CMs/ECs/MCs) exhibited highest tissue function *in vitro*, we examined the *in vivo* potential of CM+EC+MC hiPSC-ECTs to contribute to functional recovery and myocardial regeneration in an immune tolerant rat MI model (Figs 3, 4, 5, and Supplementary Videos 2,3). All implanted (n = 6) and sham operated rats (n = 5) survived the 4-week post-implant observation period with no tumor formation. We noted that implanted hiPSC-ECTs including vascular cells ameliorated left ventricular (LV) dysfunction following MI (Fig. 3c,d and Supplementary Video 3). Implanted ECTs engrafted at infarction sites and produced human nucleic antigen (HNA)^+^, thick (>800 μm) regenerated myocardium at 4 weeks after implantation (Figs 4a and 5a). All implanted rats exhibited ECT engraftment (n = 6) and the engrafted area reached 42.0 ± 16.9% of the infarcted area (ranged from 24.6% to 67.4%) (Supplementary Fig. 10). Regenerated myocardium with obvious sarcomeric structures (Fig. 4b) occupied greater than half the thickness of infarcted heart wall. We confirmed abundant von Willebrand factor (vWF)^+^ vascular luminal structures around the regenerated myocardium and penetrating to the central area of the neo-myocardium resulting in blood supply to the whole tissue (Fig. 4c). The majority of these vascular structures contained host-derived, (HNA^−^ and vWF^+^) vascular cells, consistent with host-derived vascular invasion of the implanted ECT through possible angiogenic stimuli^8^, though some vascular structures represented chimeric composites of both host and graft-derived vasculature. We further confirmed functioning vascular connection between the recipient myocardium and implanted ECT grafts using host vascular injection of fluorescent dye-conjugated lectin to identify perfused vessels within engrafted tissue (Fig. 5a). We also identified evidence for Connexin 43 expression within regenerated myocardium at 4 weeks after ECT implantation indicating existence of gap junction between CMs (Fig. 5b).

## Discussion

In the present study, we successfully generated a novel 3D ECT composition of hiPSC-derived cardiomyocytes, vascular endothelial cells, and mural cells that displays excellent *in vitro* structural maturation and electromechanical performance, is capable of *in vivo* survival, remodeling, and perfusion post-implantation, and contributes to myocardial repair and recovery. The CM+EC+MC ECT composition produced functional myocardium with structural and performance characteristics favorably suited for the altered physiology of the failing heart such as increased heart rate as a compensatory mechanism of decreased cardiac output or augmented adrenergic activity (compensatory tachycardia)^29^, and lower depolarization threshold consistent with the decreased total electrical voltage of a failing heart^30^.

As has previously been noted with the addition of fibroblasts into ECTs^25^, our approach of complementing the CM population in ECTs with vascular constituents (EC and MC) more closely resembles native myocardium and likely facilitates the maturation and the structural and vascular coupling of implanted ECTs to the recipient myocardium. Even though it remains likely that *in vitro* matured ECT remain electromechanically immature compared to native heart tissues, the co-existence of vascular constituents with CMs altered ECT biomechanical properties, including increased structural maturation and CM alignment with ECT myofibers. The functional immaturity of hiPSC-derived isolated CM and derived tissues is well recognized^16^,^31^, and the incorporation of non-CM lineages to generate more representative myocardial constructs may be one solution to this barrier to translation.

The role of non-myocytes in facilitating the *in vitro* maturation, *in vivo* survival, and adaptation of ECTs remains highly intriguing. Recognizing the complexity of quantifying gene expression patterns in this engineered construct, we noted that the addition of MCs increased transcript expression in numerous regulatory pathways associated with myocardial maturation (Fig. 2d). While, validation of the roles of individual transcripts and pathways was beyond the scope of the current study, additional experiments can explore the roles of unique cellular constituents and pathways in ECT structural and functional maturation, as well as *in vivo* survival and adaptation.

Even though the extent of functional recovery after implantation of hiPSC-derived ECTs to a rat sub-acute 1-week MI model was lower than that of our previous report of hiPSC-derived cardiac tissue sheet transplantation^8^, the implantation of ECTs showed higher efficiency of engraftment in that all 6 rats demonstrated ECT engraftment. Cell sheets potentially cover larger area of injured heart than that of ECTs which might lead to higher functional recovery especially for a pathology which might be more amended by paracrine factors, such as sub-acute MI. For the treatment of more severe pathologies resistant to indirect paracrine effects such as chronic disorders with extensive fibrosis, thicker organized 3D construct like ECTs as shown in the present study, or stacked cell sheets using gelatin hydrogel microspheres^32^ might be effective because of higher potential of engraftment which may reconstruct functioning myocardium. Further work is required to investigate the contribution of indirect paracrine factors and direct mechanical ECT support to the functional recovery after hiPSC-ECT implantation.

In the present study, we did not prepare each cardiovascular cell population using a cell sorter to avoid substantial cell loss. Our strategy was to blend multiple cell compositions with known lineage distributions to achieve the desired final lineage distribution. We successfully controlled cellular components using several non-sorting differentiation protocols by simply changing reagents and culture media during the differentiation culture to yield large number of cells available for ECT formation. This approach minimizes cell loss and avoids specimen contamination, two key issues relevant in the transition to large-animal experiments and clinical trials.

In addition to defining the therapeutic effects of hiPSC-ECTs, we need to continue to validate the safety of this therapeutic modality before conducting clinical trials^33^,^34^. All implanted rats survived the 4-week observation period without accidental death suggesting no lethal arrhythmic events occurred following ECT implantation onto vulnerable myocardium. As described in our previous report using hiPSC-derived cell sheet^8^, we need further assessment for the proarrhythmic potential of the iPSC-ECT implantation including investigation of the electrical and functional integration between the engrafted and host tissues whether the graft could work as native host tissue *in vivo* using larger animal models with similar intrinsic beating rates to the human hearts and hiPSC-derived ECTs. We also need to determine the potential for implanted hiPSC-ECTs to trigger tumor formation through longer-term studies as well as explore the generation of hiPSC-ECTs from iPSCs established with episomal plasmid vectors considered to minimize genomic integration^35^.

In conclusion, we provide new evidence that a more complex composition of hiPSC-derived cardiac cells (CM+EC+MC) incorporated into implantable bioengineered 3D ECTs shows favorable structural and functional features relevant to future clinical translation. While the optimization of hiPSC-derived CM maturation remains under intense investigation^36^, multiple lineage composition ECTs hold promise to generate hiPSC-derived cardiac tissues optimized for the failing *in situ* human myocardium. Further refinements in hiPSC-ECT compositions are likely to occur during large animal preclinical trials followed by Phase I-II clinical trials.

## Methods

Detailed methods are described in Supplementary Methods.

### Human iPSC Culture and Differentiation

Human iPSCs [4-factor (Oct3/4, Sox2, Klf4 and c-Myc) line: 201B6, previously described in detail^8^,^12^,^22^ were used for this study. In brief, iPSCs were expanded and maintained on thin-coat matrigel (growth factor reduced, 1:60 dilution; BD Biosciences, San Jose, CA) in mouse embryonic fibroblast conditioned medium (MEF-CM) supplemented with 4 ng/mL human basic fibroblast growth factor (hbFGF; WAKO, Osaka, Japan). Cells were passaged as small clusters every 4–6 days using CTK solution [0.1% collagenase IV, 0.25% Trypsin, 20% knockout serum replacement (KSR), and 1 mmol/L CaCl_2_ in phosphate buffered saline (PBS)].

Cardiovascular (CV) cell differentiation was induced as previously reported^8^,^22^ with modifications, as shown in Supplementary Fig. 1. Cells were detached following a 3 to 7 min incubation with Versene (0.48 mmol/L EDTA solution; Life Technologies, Carlsbad, CA) and seeded onto matrigel-coated plates at a density of 1,000 cells/mm^2^ in MEF-CM with 4 ng/mL hbFGF for 2 to 3 days before induction. Cells were covered with matrigel (1:60 dilution) on the day before induction. To induce CV cell population, we replaced MEF-CM with RPMI+B27 medium (RPMI1640; Life Technologies, 2 mmol/L L-glutamine; Life Technologies, 1× B27 supplement without insulin; Life Technologies) supplemented with 100 ng/mL of Activin A (R&D, Minneapolis, MN) and Wnt3a (R&D) for 24 hours (differentiation day 0; d0), followed by 10 ng/mL human bone morphogenetic protein 4 (BMP4; R&D) and 10 ng/mL hbFGF (d1) for 2 or 4 days without culture medium change. Wnt3a was used at a concentration of 100 ng/mL in all experiments unless stated otherwise. For induction of CM and EC (CM+EC protocol): The culture medium was replaced on d5 with RPMI+B27 supplemented with 50 ng/mL of VEGF_165_ (Miltenyi, Bergisch Gladbach, Germany), and culture medium was refreshed every other day. Beating cells appeared at d11 to d13. For induction of CM and MC (CM+MC protocol): The culture medium was replaced on d5. We used RPMI+B27 supplement with insulin (Life Technologies) and 100 ng/mL of Dkk1 (R&D) on d5 to 7 and refreshed culture medium every other day with RPMI+B27 supplement with insulin. For induction of MC (MC protocol): The culture medium was replaced on d3 with RPMI+10% FBS medium [RPMI1640, 2 mmol/L L-glutamine, 10% fetal bovine serum (FBS)], and culture medium was refreshed every other day.

### Human iPS Cell-Derived Engineered Cardiac Tissue (hiPSC-ECT) Formation

We combined hiPSC-derived CV cells from 3 protocols (CM+EC protocol, CM+MC protocol and MC protocol) (Fig. 1) to generate the 3 types of ECTs containing the distribution of cardiomyocytes (CM), endothelial cells (EC), and/or mural cells (MC) shown in Fig. 1a. Cells at differentiation (d15) were dissociated by incubation with Accumax (Innovative Cell Technologies, San Diego, CA). Collected cells (approximately 3.0 × 10^6^ cells/ECT) were mixed with acid-soluble rat-tail collagen type I (Sigma) and matrix factors (Matrigel; BD Biosciences) similar to our previously published methods^13^,^14^. Cell/matrix mixture was performed as follows. (1) Cells were suspended within a culture medium (high glucose-modified Dulbecco’s essential medium; Life Technologies) containing 20% fetal bovine serum (Life Technologies). (2) Acid-soluble collagen type I solution (pH 3) was neutralized with alkali buffer (0.2 M NaHCO_3_, 0.2 M HEPES, and 0.1 M NaOH) on ice. (3) Matrigel (15% of total volume) was added to the neutralized collagen solution. (4) Cell suspension and matrix solution were mixed with a final collagen type I concentration of 0.67 mg/mL. Cylindrical hiPSC-ECTs were constructed using a collagen type I-coated silicone membrane 6-well culture plate (TissueTrain; Flexcell International, Hillsborough, FL) and FX-5000TT system (Flexcell International). Briefly, the center of the silicone membrane of a TissueTrain culture plate was deformed by vacuum pressure to form a 20-mm-length × 2-mm-width trough using a cylindrical loading post (FX-5000TT). Approximately 200  μL of cell/matrix mixture was poured into the trough and incubated for 120 min in a standard CO_2_ incubator (37 °C, 5% CO_2_) to form a cylindrical construct. Both ends of the construct were held by nylon mesh anchors attached to the TissueTrain culture plate. Once the tissue gelled, the culture plate was filled with pre-culture medium [PM; alpha minimum essential medium (αMEM; Life Technologies) supplemented with 10% FBS, 5 × 10^−5^ M 2-mercaptoethanol (Sigma) and 100 U/mL Penicillin-Streptomycin (Life Technologies)]. Constructed hiPSC-ECTs were cultured for >14 days with medium change every other day.

### Contractile Force Measurements

As previously described for chick and rat ECTs^13^,^14^, we excised a central 10 mm to 15 mm length ECT segment and preserved the specimen in cold (25 °C) Tyrode’s solution containing (in mmol/L) 119.8 NaCl, 5.4 KCl, 2.5 CaCl_2_, 1.05 MgCl_2_, 22.6 NaHCO_3_, 0.42 NaH_2_PO_4_, 0.05 Na_2_EDTA, 0.28 ascorbic acid, 5.0 glucose, and 30 2,3-butanedione monoxime (BDM) gassed with 95% O_2_ and 5% CO_2_. One end of the ECT was gently attached to a force transducer (model 403A, Aurora Scientific, Ontario, Canada) and the other end to a high-speed length controller (model 322C, Aurora Scientific) mounted on a micromanipulator. Attachments were made using 10–0 nylon threads. The perfusion chamber containing the construct was then filled with BDM-free warmed Tyrode’s solution (37 °C, 1 ml total volume). During a 30 minute equilibration period the construct was field-stimulated (2 Hz/5V) (Supplementary Fig. 2b and C) and then the segment length of the tissue was gradually increased until total force reached maximum (*Lmax*) and then used *Lmax* during all subsequent force measurements. The external diameters of the tissue at each stretch increment were recorded using a microscope camera (model MU1000, AmScope, Irvine, CA), and the cross-sectional area (CSA, mm^2^) was calculated by assuming circular geometry. We determined passive and active force and relaxation time^24^ (time for 90% decrease of maximum) during pacing from 1 Hz to the paced maximum capture rate in pacing increments of 0.5 Hz. We then calculated minimum excitation threshold (ET) during 2Hz pacing. Stress values (active and passive stress; mN/mm^2^) were calculated by force values divided by the CSA of each specimen. Young’s moduli were calculated from plots of strain and passive stress.

### Animal Model Preparation and ECT Implantation

All animal surgeries were performed in accordance with protocols approved by the University of Louisville and *the Guide for the Care and Use of Laboratory Animals* prepared by the Institute for Laboratory Animal Research, USA (8th ed., 2011). Male athymic nude rats (NTac:NIH-Foxn1^rnu^, Taconic Biosciences, Hudson, NY) weighing 300–360 g were used as recipients for hiPSC-ECT transplantation. Myocardial infarction (MI) model rats were created by permanent left anterior descending artery ligation using a 7–0 silk suture as previously described^8^. Isoflurane (3–5%) inhalation was used for general anesthesia, and subcutaneous injection of Buprenorphine (0.5 mg/kg, twice a day, 3 days including operation day) was used for analgesia. ECT implantation was performed 1 week after MI induction during the “sub-acute phase” of MI. A total of 11 rats were randomly divided into two groups: rats implanted with ECTs (Tx group; n = 6) and sham-operated rats (sham group; n = 5). As previously described^13^, the LV anterior wall was exposed through left thoracotomy. Using 7–0 silk sutures, the anterior infarcted myocardium was covered with 3 ECT constructs along the LV circumferential direction (Fig. 3b and Supplementary Video 2). The pericardium was removed and ECTs were implanted onto the epicardium. For the sham-operated group, a thoracotomy was performed 1 week after coronary ligation; however, no ECT implantation was performed.

### Cardiomyocyte Alignment and Distribution Analysis

We compared CM alignment among the 3 ECT groups based on the cTnT immunostained images. Five 40× images, sampled from 3 to 5 constructs, were used per ECT group. In addition, we compared alignment to 2-dimensional (2D) cultured CM. Local CM orientation was computed from the image gradient^37^ and we computed the concentration parameter (*κ*) for the associated von Mises distribution as a measure of alignment. A larger *κ* value indicates that CM orientation is more concentrated along a single direction^38^. Analysis was carried out in Matlab^39^ (MathWorks, Natick, MA).

### Next-generation RNA sequencing

Next-generation RNA sequencing (RNA-seq) was performed at University of Louisville Center for Genetics and Molecular Medicine on the Illumina NextSeq 500 (Illumina, San Diego, CA) using NextSeq500 High Output Kits (75 cycles, Cat# FC-404-1005). Libraries were prepared using True Seq Stranded Total RNA LT Sample Prep Kit-Set B (Cat# RS-122-2302) with Ribo-Zero Gold (Illumina)^40^. Quality was checked on an Agilent Bioanalyzer using Agilent DNA 1000 Kits. The final sample fragment sizes were approximately 260 bp. Sequencing library quantitation was done by qPCR using KAPA Library Quantitation Kit for Illumina Platforms before pooling equal amounts of libraries.

The samples were then divided into 36 fastq single-end raw sequencing files representing three ECT conditions: CM+EC, CM+EC+MC, and CM+MC. Each of four single-end raw .fastq files for each replicate was concatenated into one single-end .fastq file using the Unix cat command resulting in 9 single .fastq files representing 3 conditions with 3 biological replicates. Quality control of these raw sequencing data was performed using *FastQC* (version 0.10.1). Base trimming of these raw data was performed using Trimmomatic (version 0.27) when the average quality within a window of 3 based fell below a phred score of 20. The trimmed sequences were then directly aligned to the human hg19 reference genome assembly using *tophat2* (version 2.0.13). Aligned RNA-seq reads were assembled according to the *hg19.gtf* annotation file using *cufflinks* (version 2.2.1) and the differentially expressed genes (DEGs) were identified for pairwise comparison using the tuxedo suite of programs in *cufflinks* and *cuffdiff* (version 2.2.1). DEGs at each time point with a p-value cutoff of 0.01 were used for further analysis of enriched Gene Ontology Biological Processes (GO:BP)^41^. Data were uploaded to MetaCoreTM for enrichment analysis (GeneGo, Thomson Reuters, New York, NY) and analyzed using an enrichment settings threshold of 1.5, P < 0.01.

### Statistics

The data were processed using JMP software for Windows (version10.0.2, SAS Institute Inc., Cary, NC). Comparisons between two groups were made with the unpaired t-test. Comparisons between >2 groups were made with one-way or two-way repeated analysis of variance (ANOVA) followed by Tukey’s test as post hoc. Single regression analyses were performed to evaluate correlation between 2 values. Values are shown as mean ± SD. P values < 0.05 were considered significant.

## Additional Information

**How to cite this article**: Masumoto, H. *et al*. The myocardial regenerative potential of three-dimensional engineered cardiac tissues composed of multiple human iPS cell-derived cardiovascular cell lineages. *Sci. Rep.* **6**, 29933; doi: 10.1038/srep29933 (2016).

## Supplementary Material

Supplementary Information

Supplementary Video 1

Supplementary Video 2

Supplementary Video 3

Supplementary Dataset 1

Supplementary Dataset 2

Supplementary Dataset 3

Supplementary Dataset 4

Supplementary Dataset 5

Supplementary Dataset 6
