# Supplementary material for: The myocardial regenerative potential of three-dimensional engineered cardiac tissues composed of multiple human iPS cell-derived cardiovascular cell lineages
Source: Sci Rep. 2016 Jul 20;6:29933. doi: 10.1038/srep29933 (PMC4951692; doi:10.1038/srep29933)
Supplement: Supplementary Information [file srep29933-s1.pdf]

Supporting Information

The myocardial regenerative potential of three-dimensional engineered cardiac tissues composed of multiple human iPS cell-derived cardiovascular cell lineages

Hidetoshi Masumoto[1,2,3 †], Takeichiro Nakane[1,2,3 †], Joseph P. Tinney[1,4], Fangping Yuan[1,4], Fei Ye, [1,4], William J. Kowalski[1,4], Kenji Minakata[3], Ryuzo Sakata[3], Jun K. Yamashita[2], Bradley B. Keller[1,4*]

[1] Kosair Charities Pediatric Heart Research Program, Cardiovascular Innovation Institute, University of Louisville, Louisville, Kentucky, The United States of America

[2] Department of Cell Growth and Differentiation, Center for iPS Cell Research and Application (CiRA), Kyoto University, Kyoto, Japan

[3] Department of Cardiovascular Surgery, Kyoto University Graduate School of Medicine, Kyoto, Japan

[4] Department of Pediatrics, University of Louisville School of Medicine, Louisville, Kentucky, The United States of America

*Corresponding author

[†] These authors equally contributed to this work.

Supplementary Methods

Human iPSC Culture and Differentiation

Human iPSCs [4-factor (Oct3/4, Sox2, Klf4 and c-Myc) line: 201B6, previously described in detail[1-3] were used for this study. In brief, iPSCs were expanded and maintained on thin-coat matrigel (growth factor reduced, 1:60 dilution; BD Biosciences, San Jose, CA) in mouse embryonic fibroblast conditioned medium (MEF-CM) supplemented with 4 ng/mL human basic fibroblast growth factor (hbFGF; WAKO, Osaka, Japan). Cells were passaged as small clusters every 4–6 days using CTK solution [0.1% collagenase IV, 0.25% Trypsin, 20% knockout serum replacement (KSR), and 1 mmol/L $CaCl_2$ in phosphate buffered saline (PBS)].

Cardiovascular (CV) cell differentiation was induced as previously reported[2, 3] with modifications, as shown in Figure 1. Cells were detached following a 3 to 7 min incubation with Versene (0.48mmol/L EDTA solution; Life Technologies, Carlsbad, CA) and seeded onto matrigel-coated plates at a density of 1,000 cells/mm^2 in MEF-CM with 4 ng/mL hbFGF for 2 to 3 days before induction. Cells were covered with matrigel (1:60 dilution) on the day before induction. To induce CV cell population, we replaced MEF-CM with RPMI+B27 medium (RPMI1640; Life Technologies, 2mmol/L L-glutamine; Life Technologies, 1× B27 supplement without insulin; Life Technologies) supplemented with 100 ng/mL of Activin A (R&D, Minneapolis, MN) and Wnt3a (R&D) for 24 hours (differentiation day 0; d0), followed by 10 ng/mL human bone morphogenetic protein 4 (BMP4; R&D) and 10 ng/mL hbFGF (d1) for 2 or 4 days without culture medium change. Wnt3a was used at a concentration of 100 ng/mL in all experiments unless stated otherwise. For induction of CM and EC (CM+EC protocol): The culture medium was replaced on d5 with RPMI+B27 supplemented with 50ng/mL of $VEGF_{165}$ (Miltenyi, Bergisch Gladbach, Germany), and culture medium was refreshed every other day. Beating cells appeared at d11 to d13. For induction of CM and MC (CM+MC protocol): The culture medium was replaced on d5. We used RPMI+B27 supplement with insulin (Life Technologies) and 100 ng/mL of Dkk1 (R&D) on d5 to 7 and refreshed culture medium every other day with RPMI+B27 supplement with insulin. For induction of MC (MC protocol): The culture medium was replaced on d3 with RPMI+10% FBS medium [RPMI1640, 2mmol/L L-glutamine, 10% fetal bovine serum (FBS)], and culture medium was refreshed every other day.

Flow Cytometry

HiPSC-derived CV cells were dissociated by incubation with Accumax (Innovative Cell Technologies, San Diego, CA) and stained with one or a combination of the following surface markers: anti-VCAM1 conjugated with allophyco-cyanin (APC), clone STA, 1:200 (BioLegend, San Diego, CA); anti-PDGFRβ conjugated with phycoerythrin (PE), clone 28d4, 1:100 (BD, Franklin Lakes, NJ); anti-VE-cadherin conjugated with fluorescein isothiocyanate (FITC), clone 55-7h1, 1:100 (BD). To eliminate dead cells, cells were stained with the LIVE/DEAD fixable Aqua dead cell staining kit (Life Technologies). For cell surface markers, staining was carried out in PBS with 5% FBS. For intracellular proteins, staining was carried out on cells fixed with 4% paraformaldehyde (PFA) in PBS. Cells were stained with the anti-cardiac isoform of Troponin T (cTnT) (clone 13211, Thermo Fisher Scientific, Waltham, MA) labeled with Alexa-488 using Zenon technology (Life Technologies) (1:50). The staining was performed in PBS with 5% FBS and 0.75% Saponin (Sigma, St. Louis, MO). Stained cells were analyzed on an LSRII flow cytometer (BD). Data were collected from at least 5,000 events. Data were analyzed with DIVA software version 6.1.3 (BD).

Human iPS Cell-Derived Engineered Cardiac Tissue (hiPSC-ECT) Formation

We combined hiPSC-derived CV cells from protocols 1, 2, and 3 (Supplementary Fig. 1) to generate the 3 types of ECTs containing the distribution of cardiomyocytes (CM), endothelial cells (EC), and/or mural cells (MC) shown in Fig. 1a. Cells

at differentiation (d15) were dissociated by incubation with Accumax (Innovative Cell Technologies). Collected cells (approximately 3.0×10^6 cells/ECT) were mixed with acid-soluble rat-tail collagen type I (Sigma) and matrix factors (Matrigel; BD Biosciences) similar to our previously published methods[4, 5]. Cell/matrix mixture was performed as follows. (1) Cells were suspended within a culture medium (high glucose-modified Dulbecco's essential medium; Life Technologies) containing 20% fetal bovine serum (Life Technologies). (2) Acid-soluble collagen type I solution (pH 3) was neutralized with alkali buffer (0.2M $NaHCO_3$, 0.2M HEPES, and 0.1M NaOH) on ice. (3) Matrigel (15% of total volume) was added to the neutralized collagen solution. (4) Cell suspension and matrix solution were mixed with a final collagen type I concentration of 0.67 mg/mL. Cylindrical hiPSC-ECTs were constructed using a collagen type I-coated silicone membrane 6-well culture plate (TissueTrain; Flexcell International, Hillsborough, FL) and FX-5000TT system (Flexcell International). Briefly, the center of the silicone membrane of a TissueTrain culture plate was deformed by vacuum pressure to form a 20-mm-length×2-mm-width trough using a cylindrical loading post (FX-5000TT). Approximately 200 μL of cell/matrix mixture was poured into the trough and incubated for 120 min in a standard CO_2 incubator (37℃, 5% CO_2) to form a cylindrical construct. Both ends of the construct were held by nylon mesh anchors attached to the TissueTrain culture plate. Once the tissue gelled, the culture plate was filled with pre-culture medium [PM; alpha minimum essential medium (αMEM; Life Technologies) supplemented with 10% FBS, 5×10^{-5}M 2-mercaptoethanol (Sigma) and 100U/mL Penicillin-Streptomycin (Life Technologies)]. Constructed hiPSC-ECTs were cultured for >14 days with medium change every other day.

Contractile Force Measurements

As previously described for chick and rat ECTs[4, 5], we excised a central 10mm to 15mm length ECT segment and preserved the specimen in cold (25°C) Tyrode's solution containing (in mmol/L) 119.8 NaCl, 5.4 KCl, 2.5 $CaCl_2$, 1.05 $MgCl_2$, 22.6 $NaHCO_3$, 0.42 NaH_2PO_4, 0.05 Na_2EDTA, 0.28 ascorbic acid, 5.0 glucose, and 30 2,3-butanedione monoxime (BDM) gassed with 95% O_2 and 5% CO_2. One end of the ECT was gently attached to a force transducer (model 403A, Aurora Scientific, Ontario, Canada) and the other end to a high-speed length controller (model 322C, Aurora Scientific) mounted on a micromanipulator. Attachments were made using 10-0 nylon threads. The perfusion chamber containing the construct was then filled with BDM-free warmed Tyrode's solution (37°C, 1 ml total volume). During a 30 minute equilibration period the construct was field-stimulated (2 Hz / 5V) (Supplementary Fig. 3b,c) and then the segment length of the tissue was gradually increased until total force reached maximum (*Lmax*) and then used *Lmax* during all subsequent force measurements. We measured effects of 0, 0.625, 1.25, 2.5 and 5.0mM extracellular Ca^{2+} (without pacing or 2Hz/5V pacing) on active force at *Lmax*, and function of sarcoplasmic reticulum with Caffeine (5mM) on active force at *Lmax* without pacing. EC50 (half maximal effective concentration) was calculated using spline curve calculated from active force on each Ca^{2+} concentration points as previously reported[6]. Drugs are administrated to the perfusion chamber directly. The external diameters of the tissue at each stretch increment were recorded using a microscope camera (model MU1000, AmScope, Irvine, CA), and the cross-sectional area (CSA, mm^2) was calculated by assuming circular geometry. We determined passive and active force and relaxation time[7] (time for 90% decrease of maximum) during pacing from 1 Hz to the paced maximum capture rate in pacing increments of 0.5 Hz. We then calculated minimum excitation threshold (ET) during 2Hz pacing. Stress values (active and passive stress; mN/mm^2) were calculated by force values divided by the CSA of each specimen. Young's moduli were calculated from plots of strain and passive / active stress, respectively.

Animal Model Preparation, Transplantation, and Imaging

All animal surgeries were performed in accordance with protocols approved by the University of Louisville Institutional Animal Care and *the Guide for the Care and Use of Laboratory Animals* prepared by the Institute for Laboratory Animal Research, U.S.A. (8th ed., 2011). Male athymic nude rats (NTac:NIH-Foxn1^{rnu}, Taconic Biosciences, Hudson, NY) weighing 300-360g were used as recipients for hiPSC-ECT transplantation. Myocardial infarction (MI) model rats were created by permanent left anterior descending artery ligation using 7-0 silk suture as previously described[2]. Isoflurane (3-

5%) inhalation was used for general anesthesia, and subcutaneous injection of Buprenorphine (0.5mg/kg, twice a day, 3 days including operation day) was used for analgesia. ECT implantation was performed 1 week after MI induction during the "sub-acute phase" of MI. The sub-acute phase represents an optimal transplantation window between MI related acute inflammation and MI related fibrotic scar formation, that is, the period that avoids damaging the transplanted cell with acute inflammation and obtains sufficient tissue repair before fibrotic scar formation in the chronic stage[2, 8, 9]. A total of 11 rats were randomly divided into two groups: rats implanted with ECTs (Tx group; n=6) and sham-operated rats (sham group; n=5). As previously described[4], the LV anterior wall was exposed through left thoracotomy. Using 7-0 silk sutures, the anterior infarcted myocardium was covered with 3 ECT constructs along the LV circumferential direction (Fig. 3b and Supplementary Video 2). The pericardium was removed and ECTs were implanted onto the epicardium. For the sham-operated group, a thoracotomy was performed 1 week after coronary ligation; however, no ECT implantation was performed.

Transthoracic echocardiogram was performed by an investigator blinded to group assignment using a high resolution Vevo2100 system (VisualSonics, Toronto, Canada) and 21-MHz imaging transducer (MS250; VisualSonics). Evaluations were performed before ECT implantation (6 days after MI induction), and 2 and 4 weeks after implantation[4]. Ejection fraction was calculated by single plane area-length method in long axis.

Histological Analysis

ECTs were fixed in 4% PFA and embedded with paraffin. Hearts were arrested in diastole using 200 µl of chilled 3M KCl and 0.1 M CdCl solution followed by 4% PFA (perfusion fixation), embedded in OCT compound (Sakura Finetek Japan, Tokyo, Japan) and frozen. Sequential sections from ECTs (4 µm thickness) and hearts (8 µm thickness) were prepared and stained with Masson-trichrome. For immunofluorescence staining, sections were treated with 0.01 mol/L sodium citrate buffer (pH 6.0) at 100ºC for 15 minutes and incubated with primary antibodies overnight at 4ºC; Alexa Fluor 488/594 conjugated-donkey anti mouse, donkey anti-rabbit and donkey anti-rat (1:100 to 1:400, Life Technologies) were used as secondary antibodies. Engrafted human cells in ECTs were detected by human nuclear antibody (HNA) (mouse monoclonal, clone 235-1; Millipore, Billerica, MA; 1:300). Anti-cTnT antibody (rabbit polyclonal; Abcam, Cambridge, UK; 1:400 or mouse monoclonal; clone 13211, Thermo Fisher Scientific; 1:400) was used for double staining with cTnT and HNA. The following antibodies were used for primary antibodies: anti-von Willebrand factor (vWF) (DAKO, Carpinteria, CA; 1:50); ant-Connexin 43 (Sigma; 1:100). Nuclei were visualized with DAPI (4, 6 diamidino -2-phenylindole; Life technologies). For visualization of perfused vasculature within ECTs after implantation, DyLight 488 Labeled Lycopersicon Esculentum (Tomato) Lectin (Vector Laboratories, Burlingame, CA) was administrated from inferior vena cava with laparotomy under general anesthesia and mandatory respiration (10µg/g). After 15-20 min of systemic perfusion, the rat was euthanized. The heart was freshly harvested and embedded in OCT compound and frozen for sectioning. All immunostained sections were photographed with Nikon ECLIPSE Ti Confocal System (Nikon, Tokyo, Japan) attached to a Nikon Ti-E inverted microscope platform; images were captured at 1024×1024 pixel density with 1.4 NA using Nikon NIS Elements AR software (Nikon) to save as 12-bit raw files for further processing.

To quantify the ratio of hiPSC-ECT engraftment area to scar area, five 8µm frozen sections with 50µm interval were stained for cTnT and HNA to identify non-cardiomyocytes area and hiPSC-ECT engraftment areas, respectively. Sections adjacent to the cTnT/HNA stained slides were used for Trichrome staining to confirm the scar area. Sections were imaged under Plan Fluor 10x DIC L N1 objective using Nikon ECLIPSE Ti-E inverted microscope platform, performed by Scan Large Area tool. HiPSC-ECT engraftment area and scar area were processed using ImageJ software (National Institutes of Health, Bethesda, MD)[10] to calculate the ratio of hiPSC-ECT engraftment area to scar area (Supplementary Fig. 10).

Cardiomyocyte Alignment and Distribution Analysis

CM alignment was quantified from cTnT immunostained images of ECTs using an automated method based on image gradient[11, 12]. We analyzed five 40× images sampled from a set of 3 to 5 ECT constructs from each ECT group. For each

image, local cardiomyocyte fiber orientations (θ) were calculated (average N = 284 measurements per image). To quantify alignment, we computed the maximum likelihood estimate of the concentration parameter (κ) for the von Mises distribution of the local orientations[13]. A Rayleigh test was used to reject a uniform distribution at significance level 0.05, which was satisfied for each image[13]. Concentration κ is the solution to equation (1), where I_α is the modified Bessel function of the first kind, order α. Larger values of κ represent greater alignment to a single direction. In addition, we computed the circular standard deviation (ν) to assess the degree of alignment. Larger circular standard deviation reflects a more random orientation of CM.

$$\frac{I_1(\kappa)}{I_0(\kappa)} = \frac{1}{N}\sum \exp(i\theta) \qquad (1)$$

$$n = \sqrt{-2\ln\left(\frac{1}{N}\left|\sum \exp(iq)\right|\right)} \qquad (2)$$

To obtain local orientations, we applied the method designed by Chaudhuri et al.[12] and adapted for myocardium by Karlon et al.[11]. Image analysis and circular statistics were implemented with Matlab[14] (MathWorks, Natick, MA). For a given image, the horizontal (G_x) and vertical (G_y) intensity gradients were calculated by convolution with masks h_x and h_y, respectively:

$$h_x(i,j) = \frac{2i}{s^2}\exp\left(-\left(i^2+j^2\right)/s^2\right) \qquad (3)$$

$$h_y(i,j) = \frac{2j}{s^2}\exp\left(-\left(i^2+j^2\right)/s^2\right) \qquad (4)$$

where i and j are the x and y positions, respectively, and σ controls the spatial influence of the mask. In this study, we applied $\sigma = 3$ and a mask size of 6, such that $-6 \le i, j \le 6$. The gradient magnitude (G) and direction (Φ) were calculated from G_x and G_y.

$$G = \sqrt{G_x^2 + G_y^2} \qquad (5)$$

$$F = \tan^{-1}\left(G_y/G_x\right) \qquad (6)$$

The gradient direction points from low to high intensity. Therefore, the orientation of foreground objects in an image is perpendicular to this direction, $\phi = \Phi + 90°$. We adjusted all orientations to range from -90° to 90°. To calculate the local dominant orientation, the image was divided into subregions of size $m \times m$. For each subregion, an accumulator bin value $A^W(\theta)$ was computed for all possible angles $\theta \in$ [-90°, 90°). The accumulator bin assumes a von Mises distribution with mean direction θ and then sums the probabilities of each orientation ϕ. The largest accumulator bin thus defines the dominant orientation for the subregion.

$$A^W(q) = \sum_{i,j} G(i,j)\frac{\exp\left\{2\cos\left[2\left(q - f(i,j)\right)\right]\right\}}{\exp(2)} \qquad (6)$$

In this study, bins were quantized in 1° increments and subregion size m was 32 pixels, approximately the size of a cell nucleus. As the cTnT images can contain large regions of background, we restricted our analysis to only those subregions with a mean intensity greater than 1.5 times the global mean intensity of the image. Local dominant orientations were then adjusted such that the 0° reference axis coincided with the long axis of the ECT. ECT long axes were measured from 10× images. The κ value and circular standard deviation for the image were calculated according to (1) and (2) above. Additionally, we compared ECTs to 2-dimensional (2D) cultured cardiomyocytes. For 2D samples, we prepared 200μl of

cell/matrix mixture as the same density as used for ECT formation and plated in 24-well plates, cultured for 14 days, collected and proceeded to immunostaining as described above.

Transmission Electron Microscopy

hiPSC-ECTs were washed in phosphate buffer and fixed with 3% glutaraldehyde. Samples were post-fixed with 0.5% osmium, rinsed, dehydrated, and embedded in araldite (Durcupan; Sigma). Semi-thin sections (1.5 μm) were cut with a diamond knife and stained lightly with 1% toluidine blue. Semi-thin sections were re-embedded in an araldite block and detached from the glass slide by repeated freezing (liquid nitrogen) and thawing. Blocks with semi-thin sections were cut in ultrathin (0.05 μm) sections with a diamond knife, stained with lead citrate, and examined under a Philips CM10 Electron Microscope (Philips, Eindhoven, The Netherlands).

RNA extraction and Next-generation RNA sequencing

Fresh hiPSC-ECT samples were homogenized in Trizol using an Omnitip Tissue homogenizer (USA Scientific, Ocala, USA; Cat. No. 6615-7273) and total RNA was isolated using RNeasy Mini Kit (Qiagen, Valencia, USA; Cat. No. 74104) as previously described[15]. Total RNA was treated with DNAse I (Invitrogen Cat. No.18068-015) to remove genomic DNA. All ECT samples were from independent experiments and each experiment group included n=3 biological samples. RNA quality (purity and integrity) was assessed using OD260/280 and OD260/230 NanoDrop ND-2000 (Thermo Fisher Scientific) and RIN the Bioanalyzer 2100 (Agilent Technologies Inc., Santa Clara, USA).

Next-generation RNA sequencing (RNA-seq) was performed at University of Louisville Center for Genetics and Molecular Medicine on the Illumina NextSeq 500 (Illumina, San Diego, CA) using NextSeq500 High Output Kits (75 cycles, Cat# FC-404-1005). Libraries were prepared using True Seq Stranded Total RNA LT Sample Prep Kit-Set B (Cat# RS-122-2302) with Ribo-Zero Gold (Illumina)[16]. Quality was checked on an Agilent Bioanalyzer using Agilent DNA 1000 Kits. The final sample fragment sizes were approximately 260bp. Sequencing library quantitation was done by qPCR using KAPA Library Quantitation Kit for Illumina Platforms before pooling equal amounts of libraries.

The samples were then divided into 36 fastq single-end raw sequencing files representing three conditions: CM+EC, CM+EC+MC, and CM+MC. Each of four single-end raw .fastq files for each replicate was concatenated into one single-end .fastq file using the Unix cat command resulting in 9 single .fastq files representing 3 conditions with 3 biological replicates. Quality control of these raw sequencing data was performed using *FastQC* (version 0.10.1). Base trimming of these raw data was performed using Trimmomatic (version 0.27) when the average quality within a window of 3 based fell below a phred score of 20. The trimmed sequences were then directly aligned to the human hg19 reference genome assembly using *tophat2* (version 2.0.13). Aligned RNA-seq reads were assembled according to the *hg19.gtf* annotation file using *cufflinks* (version 2.2.1) and the differentially expressed genes (DEGs) were identified for pairwise comparison using the tuxedo suite of programs in *cufflinks* and *cuffdiff* (version 2.2.1). DEGs at each time point with a p-value cutoff of 0.01 were used for further analysis of enriched Gene Ontology Biological Processes (GO:BP)[17]. Data were uploaded to MetaCoreTM for enrichment analysis (GeneGo, Thomson Reuters, New York, NY) and analyzed using an enrichment settings threshold of 1.5, $P < 0.01$.

Statistics

The data were processed using JMP software for Windows (version10.0.2, SAS Institute Inc., Cary, NC). Comparisons between two groups were made with the unpaired t-test. Comparisons between >2 groups were made with one-way or two-way repeated analysis of variance (ANOVA) followed by Tukey’s test as post hoc. Single regression analyses were performed to evaluate correlation between 2 values. Values are shown as mean±SD. P values <0.05 were considered significant.

References

1. Takahashi K, Tanabe K, Ohnuki M, Narita M, Ichisaka T, Tomoda K and Yamanaka S. Induction of pluripotent stem cells from adult human fibroblasts by defined factors. *Cell*. 2007;131:861-72.
2. Masumoto H, Ikuno T, Takeda M, Fukushima H, Marui A, Katayama S, Shimizu T, Ikeda T, Okano T, Sakata R and Yamashita JK. Human iPS cell-engineered cardiac tissue sheets with cardiomyocytes and vascular cells for cardiac regeneration. *Sci Rep*. 2014;4:6716.
3. Uosaki H, Fukushima H, Takeuchi A, Matsuoka S, Nakatsuji N, Yamanaka S and Yamashita JK. Efficient and Scalable Purification of Cardiomyocytes from Human Embryonic and Induced Pluripotent Stem Cells by VCAM1 Surface Expression. *PLoS One*. 2011;6.
4. Fujimoto KL, Clause KC, Liu LJ, Tinney JP, Verma S, Wagner WR, Keller BB and Tobita K. Engineered fetal cardiac graft preserves its cardiomyocyte proliferation within postinfarcted myocardium and sustains cardiac function. *Tissue engineering Part A*. 2011;17:585-96.
5. Tobita K, Liu LJ, Janczewski AM, Tinney JP, Nonemaker JM, Augustine S, Stolz DB, Shroff SG and Keller BB. Engineered early embryonic cardiac tissue retains proliferative and contractile properties of developing embryonic myocardium. *American journal of physiology Heart and circulatory physiology*. 2006;291:H1829-37.
6. Stoehr A, Neuber C, Baldauf C, Vollert I, Friedrich FW, Flenner F, Carrier L, Eder A, Schaaf S, Hirt MN, Aksehirlioglu B, Tong CW, Moretti A, Eschenhagen T and Hansen A. Automated analysis of contractile force and Ca2+ transients in engineered heart tissue. *Am J Physiol Heart Circ Physiol*. 2014;306:H1353-63.
7. Morgan KY and Black LD, 3rd. Mimicking isovolumic contraction with combined electromechanical stimulation improves the development of engineered cardiac constructs. *Tissue Eng Part A*. 2014;20:1654-67.
8. Li RK, Mickle DA, Weisel RD, Rao V and Jia ZQ. Optimal time for cardiomyocyte transplantation to maximize myocardial function after left ventricular injury. *The Annals of thoracic surgery*. 2001;72:1957-63.
9. Masumoto H, Matsuo T, Yamamizu K, Uosaki H, Narazaki G, Katayama S, Marui A, Shimizu T, Ikeda T, Okano T, Sakata R and Yamashita JK. Pluripotent stem cell-engineered cell sheets reassembled with defined cardiovascular populations ameliorate reduction in infarct heart function through cardiomyocyte-mediated neovascularization. *Stem Cells*. 2012;30:1196-205.
10. Schneider CA, Rasband WS and Eliceiri KW. NIH Image to ImageJ: 25 years of image analysis. *Nat Methods*. 2012;9:671-5.
11. Karlon WJ, Hsu PP, Li S, Chien S, McCulloch AD and Omens JH. Measurement of orientation and distribution of cellular alignment and cytoskeletal organization. *Ann Biomed Eng*. 1999;27:712-20.
12. Chaudhuri BB, Kundu P and Sarkar N. Detection and Gradation of Oriented Texture. *Pattern Recognition Letters*. 1993;14:147-153.
13. Fisher NI. *Statistical analysis of circular data*. Cambridge: Cambridge University Press; 1995.
14. Berens P. CircStat: A MATLAB Toolbox for Circular Statistics. *J Stat Softw*. 2009;31:1-21.
15. Ye F, Yuan F, Li X, Cooper N, Tinney JP and Keller BB. Gene expression profiles in engineered cardiac tissues respond to mechanical loading and inhibition of tyrosine kinases. *Physiol Rep*. 2013;1:e00078.
16. Trapnell C, Hendrickson DG, Sauvageau M, Goff L, Rinn JL and Pachter L. Differential analysis of gene regulation at transcript resolution with RNA-seq. *Nat Biotechnol*. 2013;31:46-+.
17. Ashburner M, Ball CA, Blake JA, Botstein D, Butler H, Cherry JM, Davis AP, Dolinski K, Dwight SS, Eppig JT, Harris MA, Hill DP, Issel-Tarver L, Kasarskis A, Lewis S, Matese JC, Richardson JE, Ringwald M, Rubin GM and Sherlock G. Gene ontology: tool for the unification of biology. The Gene Ontology Consortium. *Nat Genet*. 2000;25:25-9.

Supplementary Figures

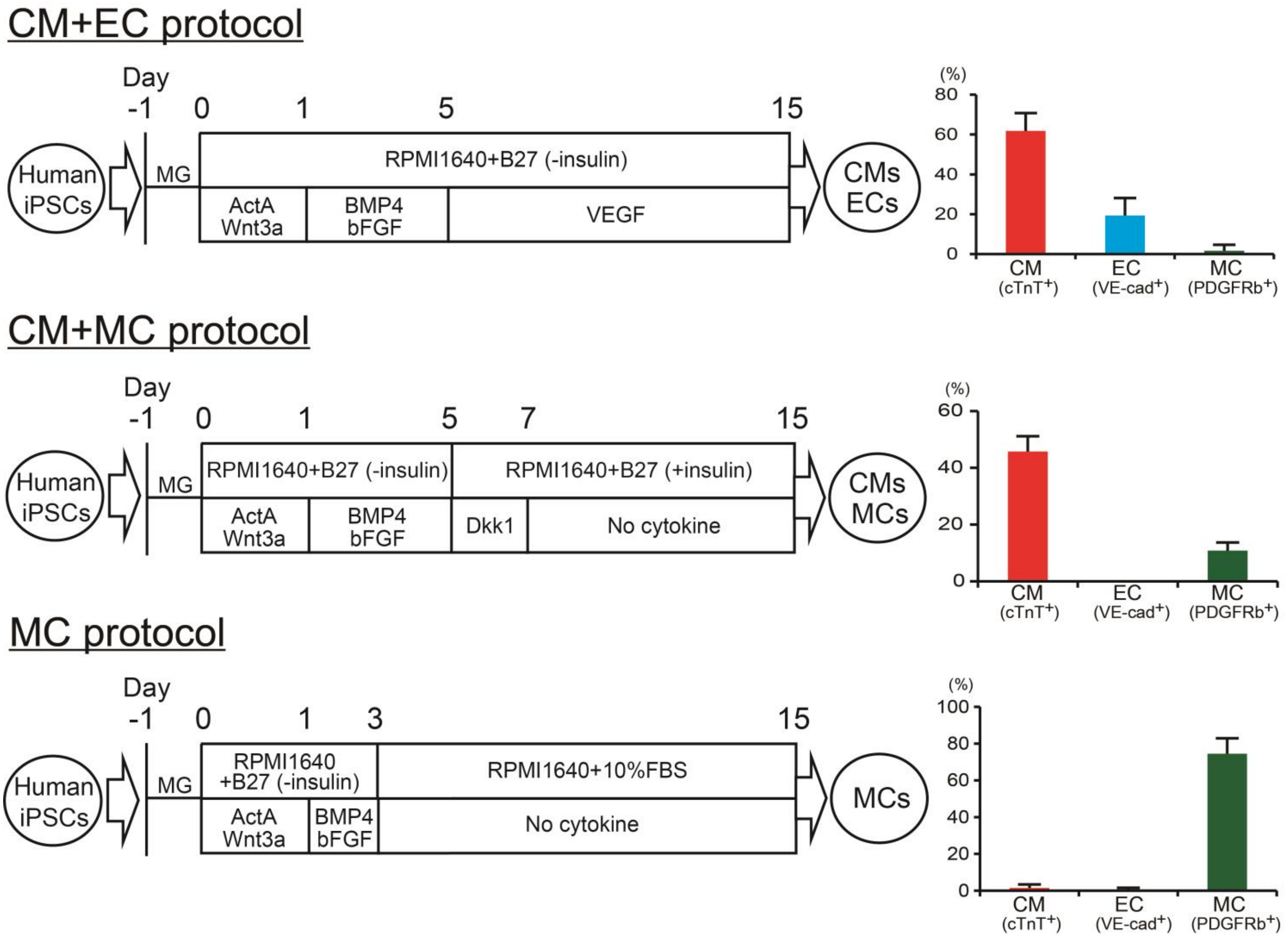

Supplementary Figure 1: Protocols to differentiate cardiovascular cell populations from human iPS cells.

Schematic diagrams of 3 different protocols (left) and flow cytometry of cell population induced by each protocol (right) for n= 26 (CM+EC protocol), n=5 (CM+MC protocol), and n=42 (MC protocol). iPSC, induced pluripotent stem cell; MG, Matrigel; ActA, Activin A; BMP4, Bone morphogenetic protein 4; bFGF, basic fibroblast growth factor; VEGF, vascular endothelial cell growth factor; CM, cardiomyocytes; EC, endothelial cell; MC, vascular mural cell; cTnT, cardiac troponin T; VE-cad, vascular endothelial cadherin; PDGFRb, platelet-derived growth factor receptor beta; Dkk1, dickkopf WNT signaling pathway inhibitor 1; FBS, fetal bovine serum.

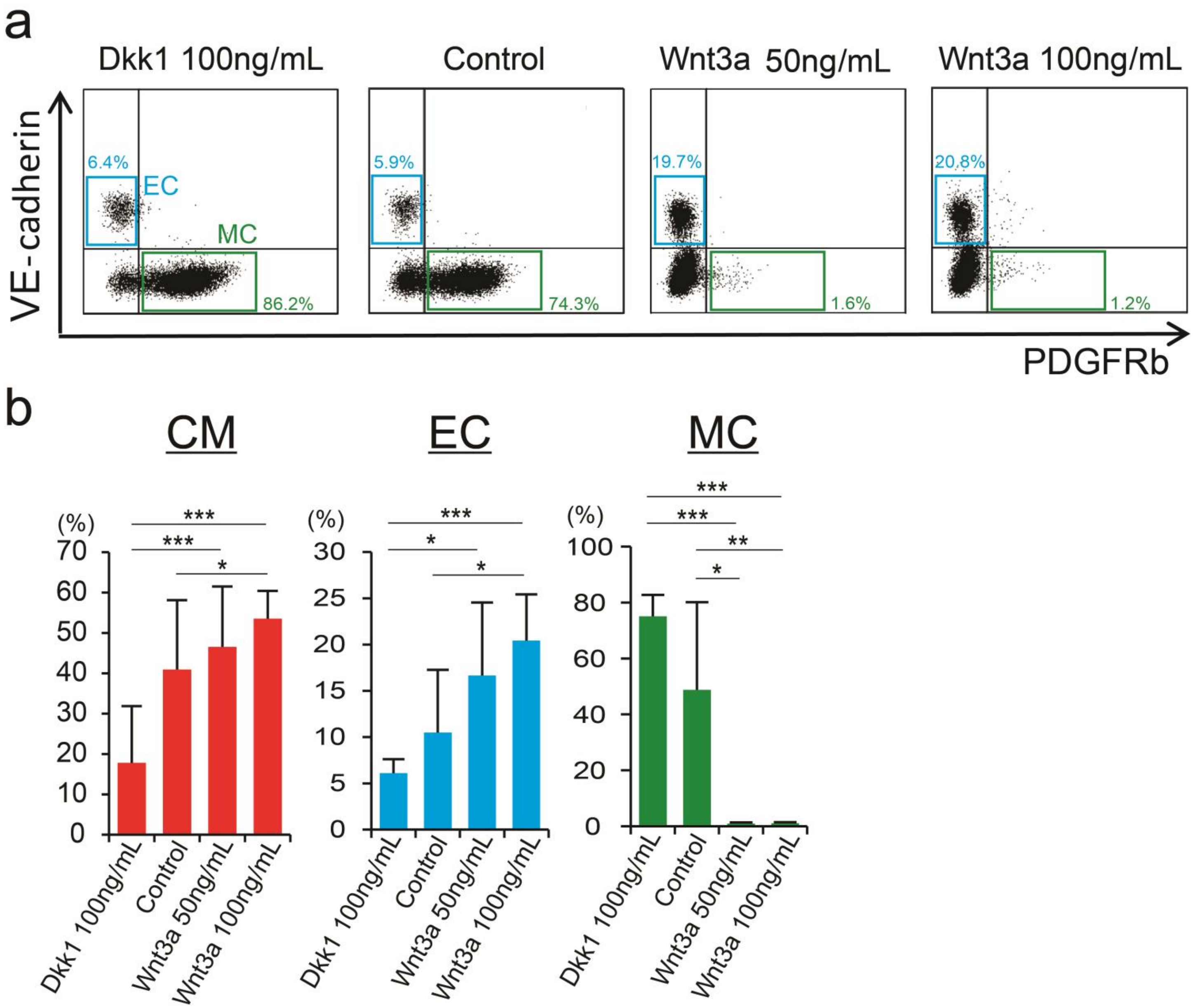

Supplementary Figure 2: Effect of Wnt3a on cardiovascular cell induction at early stage of differentiation.

(**a**) Representative dot plot of flow cytometry. Wnt3a or Dkk1 (Wnt antagonist) was administrated on differentiation day 0-1 (24hrs) expect control. (**b**) Effect of Wnt3a for CM (left), EC (center) and MC (right) induction (n=3-4). CM, cardiomyocytes; EC, endothelial cells; MC, vascular mural cells; Dkk1, dickkopf WNT signaling pathway inhibitor 1. *P<0.05, **P<0.01. ***P<0.001.

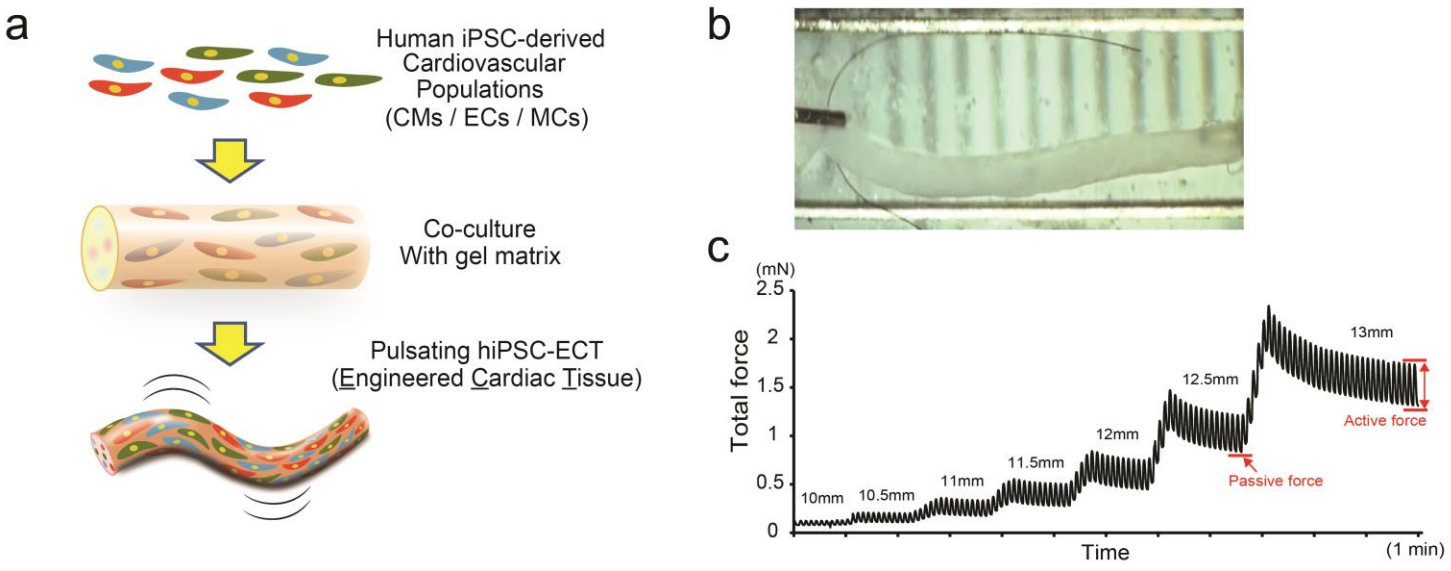

Supplementary Figure 3: hiPSC-ECT formation and contractile force measurement.

(**a**) Schematic diagram of hiPSC-ECT formation. ECT, engineered cardiac tissue. (**b**)(**c**) Contractile force measurement. (**b**) ECT attached on force transducer and length controller. (**c**) Representative force-length relationship measurement. Passive and active force at each tissue length are shown.

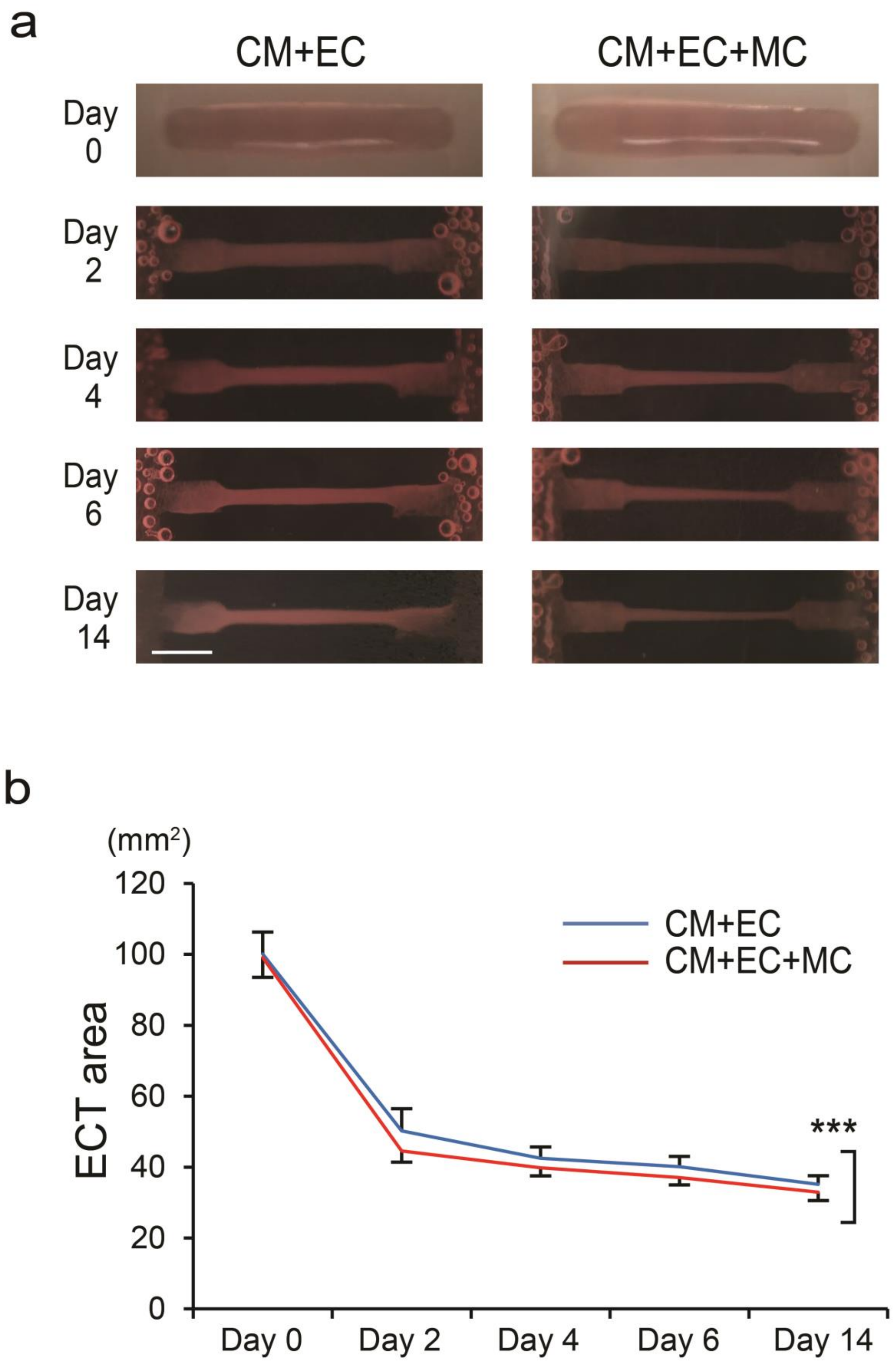

Supplementary Figure 4: Gel compaction during pre-culture of hiPSC-ECTs.

(**a**) Macroscopic view of ECTs of each type of ECT (left, C+E; right, C+E+M) along with pre-culture periods (day 0-14).
(**b**) Measured ECT area. ***P<0.001.

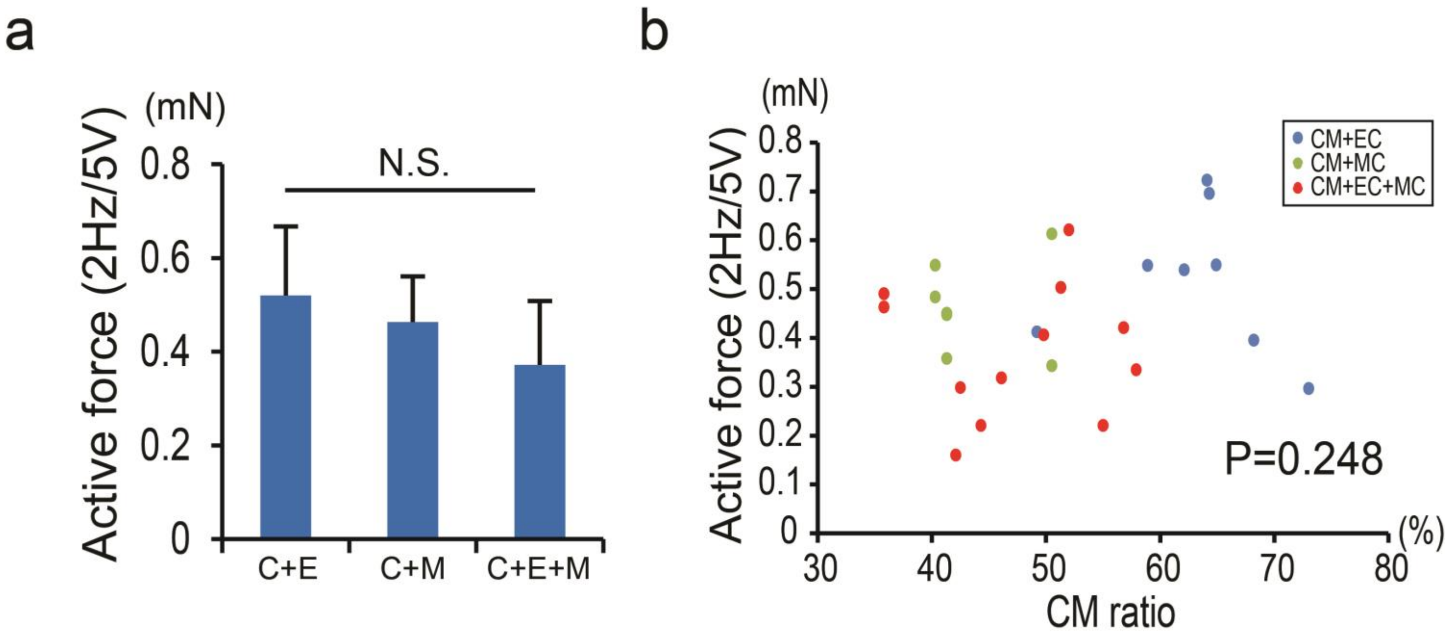

Supplementary Figure 5: Active force of hiPSC-ECTs.

(**a**) Active force of total tissue at Lmax paced at 2Hz/5V pacing. (**b**) Correlation between Active force at Lmax/2Hz/5V and CM ratio. N.S., Not significant.

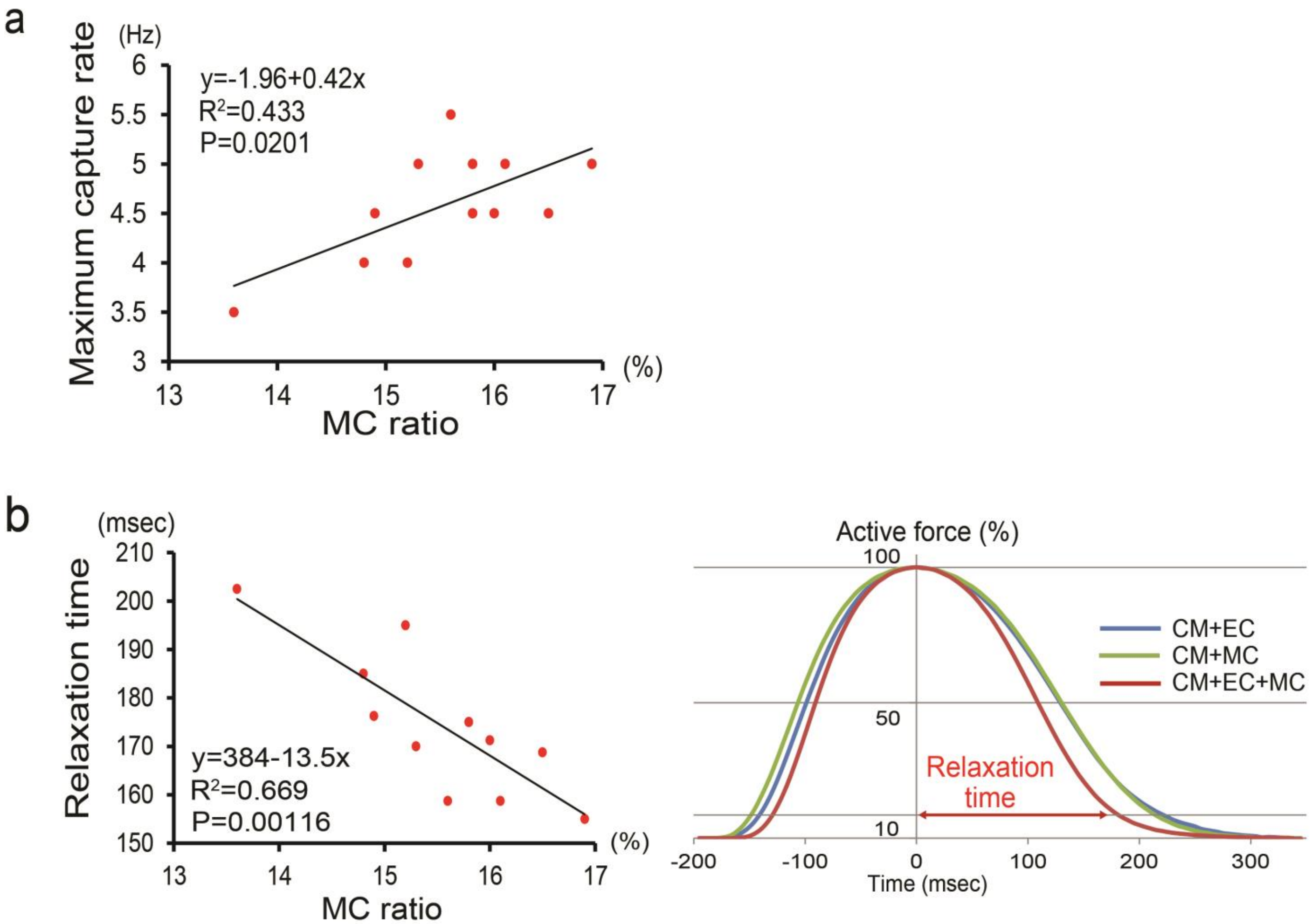

Supplementary Figure 6: Correlation between mural cell ratio and capture rate or relaxation time.

(**a**) Correlation between maximum capture rate and MC ratio. (**b**) Correlation between relaxation time and MC ratio (left). Representative relaxation transient curves with the times of 90% decrease of force (=relaxation time) identified (right).

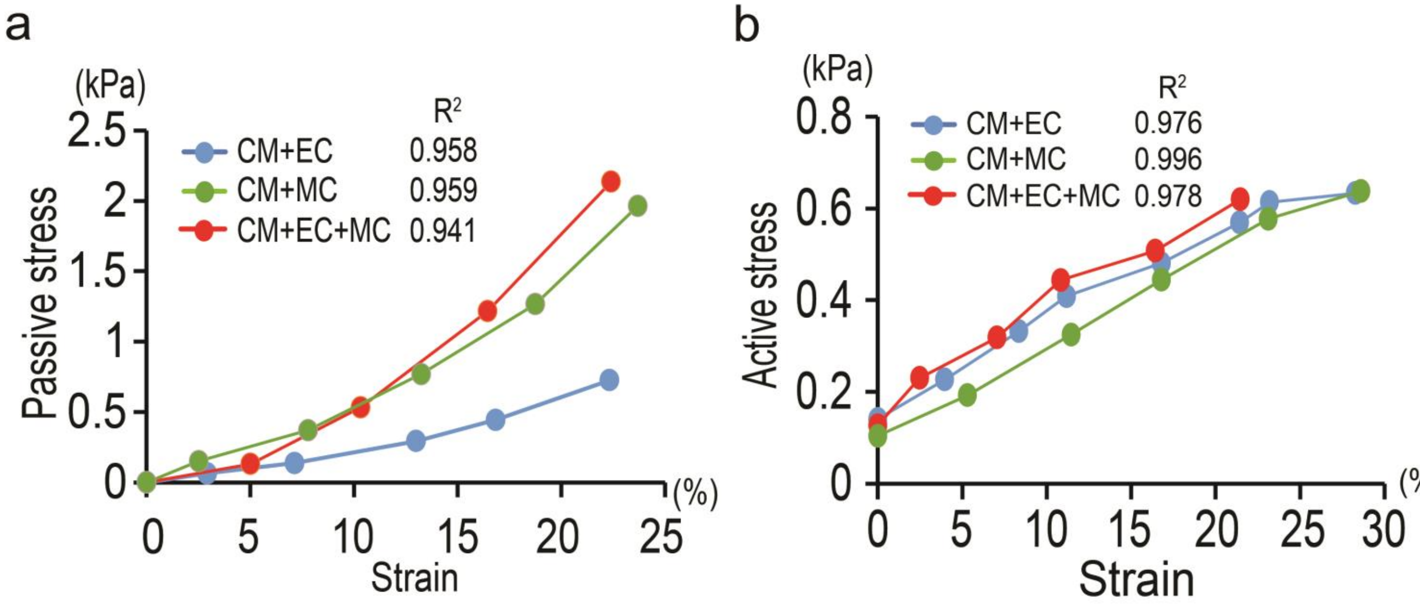

Supplementary Figure 7: Plotting of passive/active stress and strain from slack length.

(**a**) Representative plotting of passive stress and strain from slack length (up to 25%). (**b**) Representative plotting of active stress and strain from slack length (up to 30%).

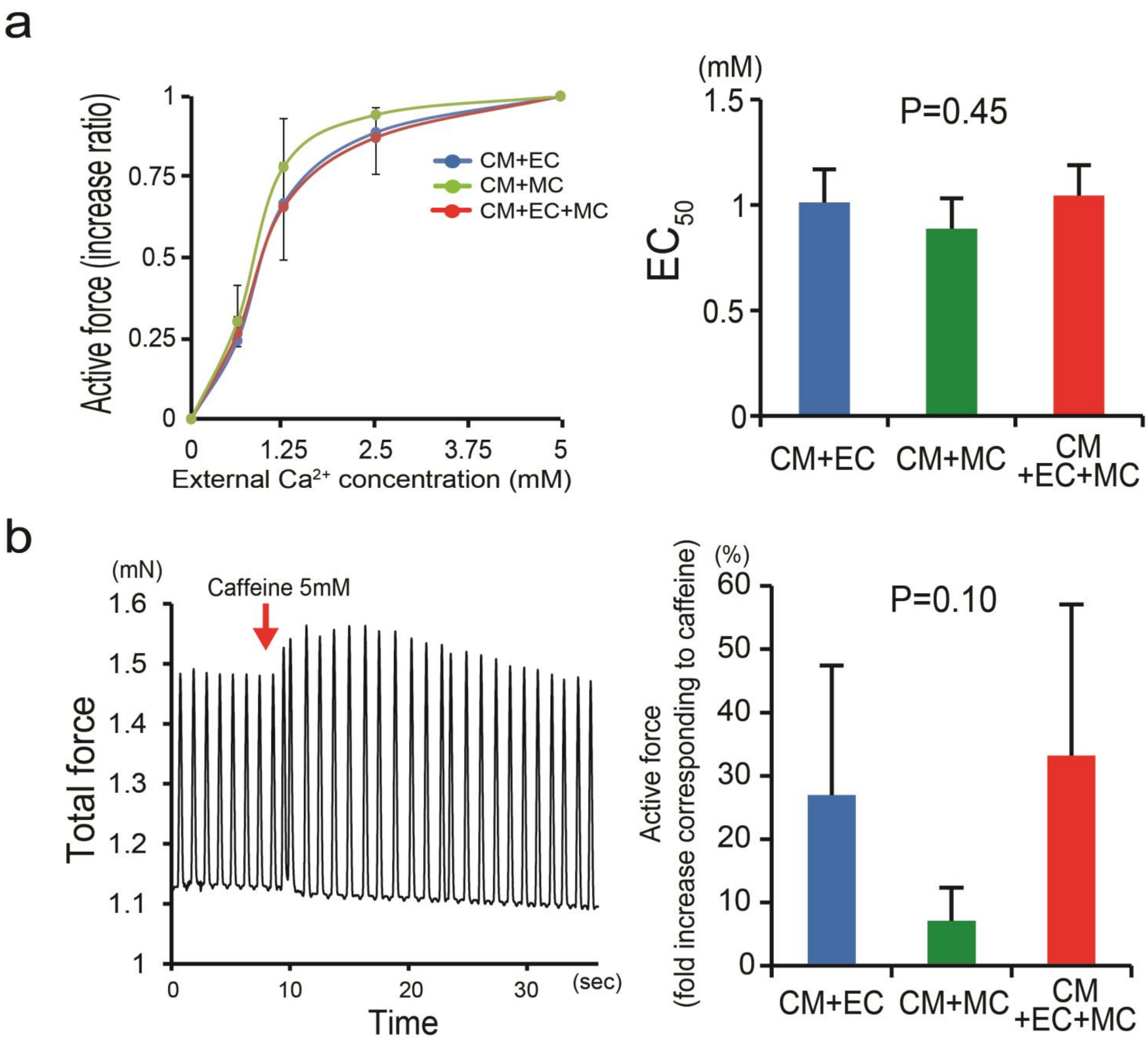

Supplementary Figure 8: Sarcoplasmic reticulum function of hiPSC-ECTs.

(a) Increase ratio of active force according to gradually increasing external Ca2+ concentration (0-5mM) (left). EC50 (half maximal effective concentration) calculated from spline curve. **(b)** Representative response in active force increase on caffeine administration (left). Fold increase of active force corresponding to 5mM caffeine administration (right).

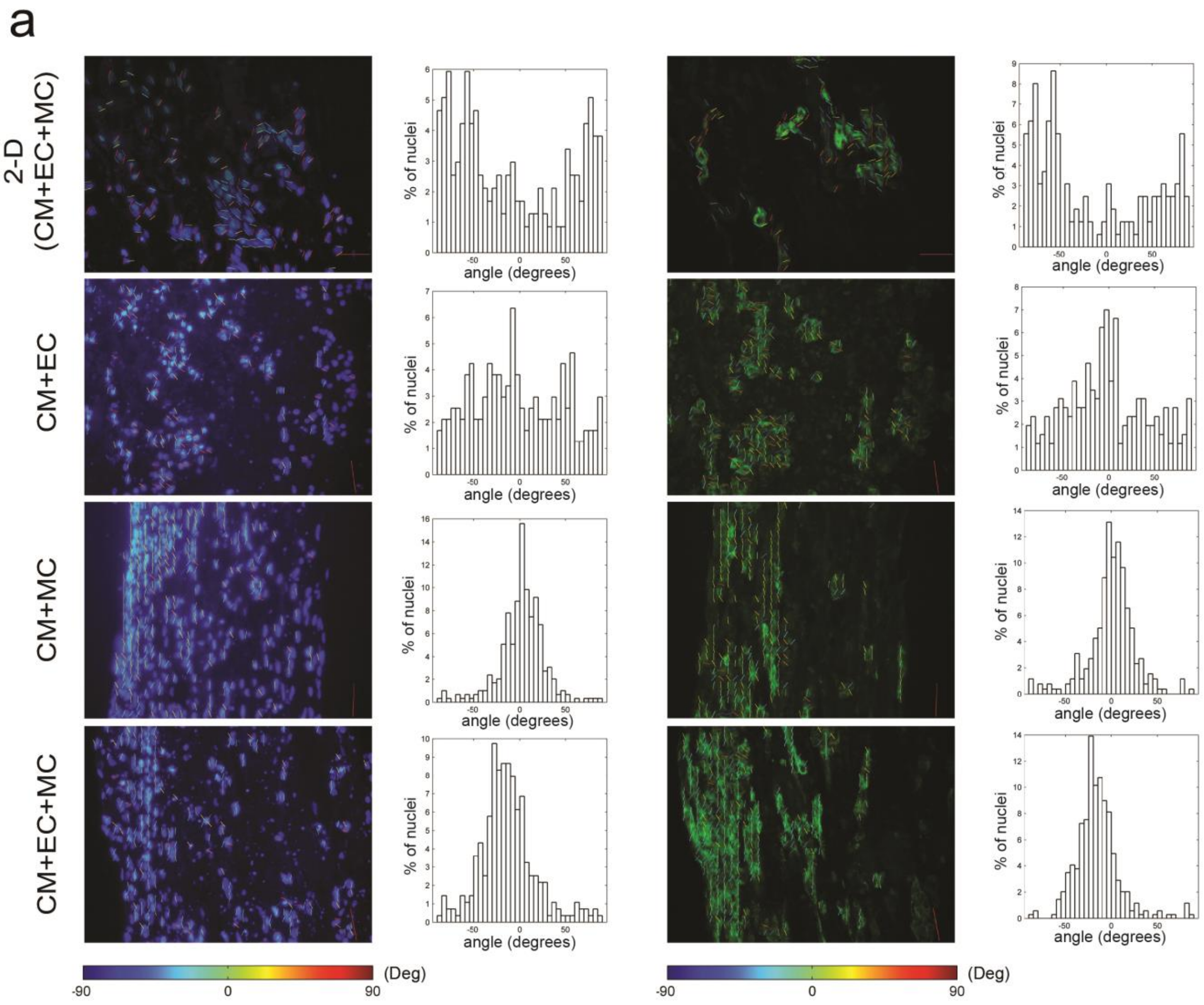

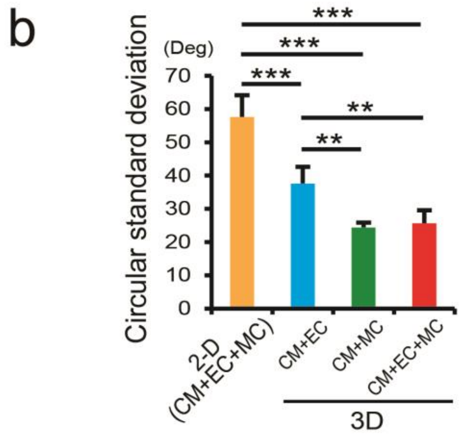

Supplementary Figure 9: CM alignment of 2D and 3D constructs.

(a) CM alignment based on nuclei (left; DAPI staining) (blue indicates nuclei) and myofiber (right; cTnT staining) (green indicates myofiber). Graphs show distribution of cellular angle of each cell to long axis of ECT (to 0 degree for 2D culture). DAPI, 4, 6 diamidino -2-phenylindole; cTnT, cardiac troponin-T. (b) Calculated circular standard deviation, an index of that is inversely related to myofiber alignment, for each culture condition (right) [n=3 (2D) and 5 (each 3D)]. **P<0.01, ***P<0.001.

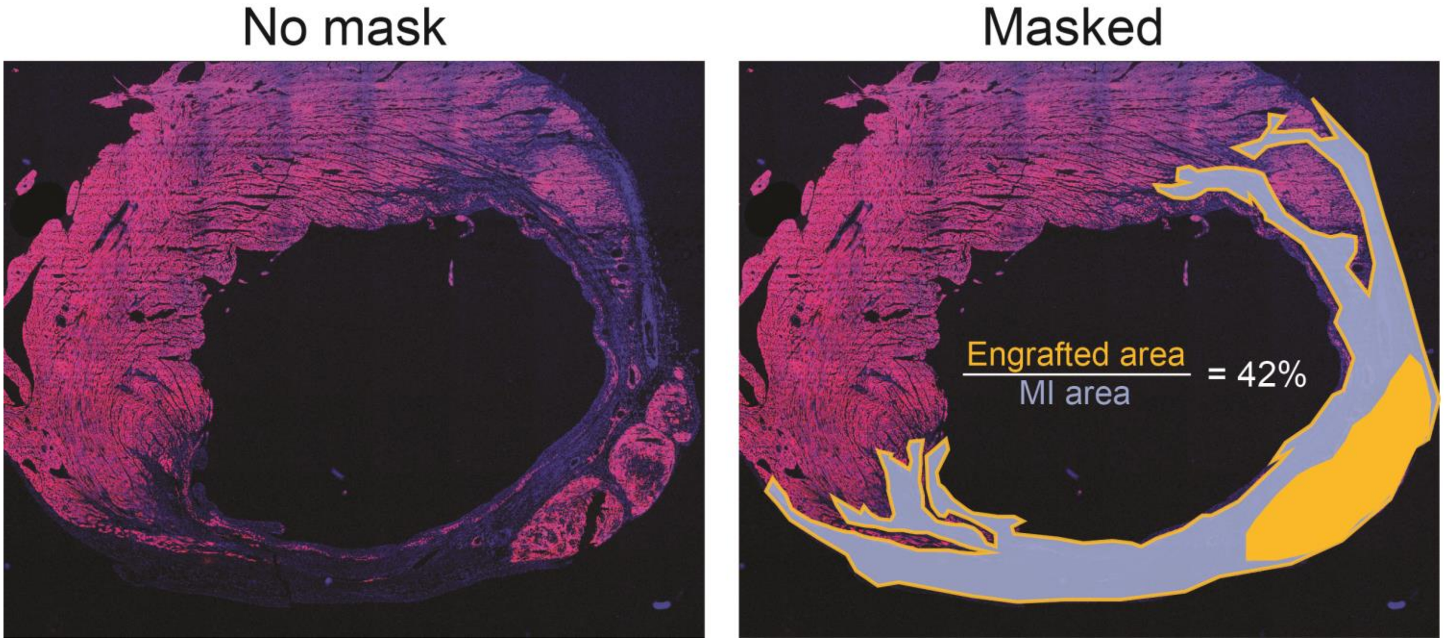

Supplementary Figure 10: Calculation for ECT engraftment.

Whole image of rat heart stained with cTnT (red) and DAPI (blue) (left; no mask) was masked to quantify the engrafted area (right; masked). Gray area indicates myocardial infarction (MI) area. Orange area indicates engrafted area. cTnT, cardiac isoform of Troponin T; DAPI, 4, 6 diamidino -2-phenylindole.

Supplementary Video legends

Supplementary Video 1: Contractile hiPSC-ECTs.

Macroscopic view in culture, microscopic view in culture and macroscopic view just after cutting off the anchors.

Supplementary Video 2: hiPSC-ECT implantation to infarcted rat heart.

Three hiPSC-ECTs bound just before implantation, and macroscopic view of rat heart just after implantation.

Supplementary Video 3: Echocardiogram after implantation.

Representative B-mode long-axis echocardiogram for rats 4 weeks after sham operation or implantation.
